# Expression profiles of transcription factors and aquaporins suggest putative roles in rubber biosynthesis regulation and drought stress adaptation in guayule

**DOI:** 10.1038/s41598-026-44868-9

**Published:** 2026-04-07

**Authors:** Huy Phan, Hussein Abdel-Haleem

**Affiliations:** US Arid Land Agricultural Research Center, USDA-Agricultural Research Services, Maricopa, AZ 85138 USA

**Keywords:** Guayule, Drought stress, Transcription factors, Aquaporin, Rubber, Plant sciences, Natural variation in plants, Plant breeding, Plant genetics, Plant stress responses

## Abstract

In arid regions of the southwestern United States, selecting drought-tolerant crops is essential for sustaining agricultural productivity and resource efficiency. Guayule (*Parthenium argentatum* Gray), a desert rubber-producing plant, utilizes complex mechanisms to adapt to water-limited environments. Understanding these strategies is important for enhancing drought resilience and maximizing rubber production. This study investigated guayule’s transcriptional response to drought stress in two developed cultivars, AZ-4 and CAL-2, selected for their contrasting responses to reduced irrigation. RNA sequencing and bioinformatics analyses revealed key transcription factors governing metabolic and stress-response pathways, highlighting distinct regulatory strategies. As indicated by GO-term enrichment and KEGG pathway analyses, under drought stress conditions, AZ-4 exhibited a more dynamic regulatory approach with more transcripts differentially regulated, emphasizing resource management and adaptive stress-response mechanisms. In contrast, CAL-2 maintained stability through precise regulatory adjustments and external defense strategies. The two cultivars demonstrated core stress-response mechanisms, particularly regulating AP2/ERF, MYB, and NAC transcription factor families for downstream regulations. Furthermore, both cultivars utilized the regulation of six key families, including AP2/ERF, bHLH, bZIP, MYB, NAC, and WRKY, that govern the MEP (methylerythritol phosphate) and MVA (mevalonate) pathways for terpenoid and natural rubber biosynthesis. However, AZ-4 displayed stronger baseline regulation of rubber biosynthesis under non-stress conditions. Additionally, aquaporin expression patterns suggested selective upregulation of PIP1-2, PIP1-4, and NIP5-1, as a compensatory mechanism for maintaining physiological functions under drought stress. These findings enhance our understanding of guayule’s drought tolerance and rubber production potential. Building on this foundation, future research should further investigate the molecular drivers of drought adaptation, leveraging multi-omics approaches to refine cultivar selection and optimize rubber yield under water-limited conditions, ultimately offering valuable help for breeding and genetic engineering strategies to improve crop performance in arid regions of the southwestern United States.

**Supplementary Information:**

The online version contains supplementary material available at 10.1038/s41598-026-44868-9.

## Introduction

Guayule (*Parthenium argentatum* Gray) is a perennial shrub native to the Southwestern United States and Northern Mexico deserts, recognized as a promising alternative source of natural rubber^1^. Unlike *Hevea brasiliensis* (Wild.) Muell.-Arg, the traditional source of natural rubber, guayule thrives in arid regions and offers resilience against potential threats, including diseases that have heavily damaged rubber tree populations in South Asia in the past^1^. Cultivating guayule can help diversify rubber production and reduce dependence on a single species, ensuring stability under environmental challenges. Its ability to grow with minimal water input highlights its potential as a sustainable crop, especially for regions facing water scarcity^2,3^. Beyond its role in the rubber industry, guayule serves as an invaluable model for studying drought tolerance and rubber production under challenging environmental conditions^4,5^. The drought-tolerant nature of guayule not only reduces its water demand compared to other crops but also supports its candidacy as a domestic natural rubber source, reducing reliance on imports^1^. Research reveals intriguing trade-offs in guayule, where increased water availability boosts biomass production, but water deficit enhances rubber content relative to biomass weight^5–10^. This adaptability highlights its efficiency in resource allocation and potential for sustainable rubber yield, even under water-limited conditions. Understanding guayule’s ability to withstand drought is crucial for optimizing its rubber production and furthering its development as a sustainable crop. Additionally, variations in physiological traits among guayule cultivars from California and Arizona reflect adaptation to distinct environments, with Arizona cultivars showing enhanced resilience to extreme heat and dryness^2,11,12^.

Identifying candidate genes which regulate drought stress tolerance could reveal key genetic and molecular mechanisms underlying their stress responses, guiding breeding efforts to enhance drought tolerance and productivity. Plants in general have evolved diverse mechanisms to adapt to environmental stresses by leveraging transcription factor (TF) families. These TFs regulate complex gene networks that allow plants to cope with both abiotic stresses, such as drought, and biotic stresses, like pathogen attacks^13–15^. Among plant TF families, the ERF, MYB, and NAC families are particularly well-studied and documented for their roles in regulating stress responses and adaptation^16–20^. ERF transcription factors play a crucial role in activating genes involved in water-use efficiency, osmotic adjustment, and stomatal regulation, making them central to drought tolerance^21–24^. MYB factors contribute to drought resilience by regulating secondary metabolism, lignin biosynthesis, root elongation, and osmolyte accumulation^25,26^, while NAC factors oversee processes like antioxidant defense, cell wall remodeling, and water retention^27–29^. Although these TFs have been extensively characterized in many plant species, examining their roles in guayule can confirm whether these stress tolerance mechanisms apply and identify traits particular to guayule.

Furthermore, in guayule, production of natural rubber is fundamentally linked to the biosynthesis of terpenes and terpenoids, which originate from the universal five-carbon precursor isopentenyl pyrophosphate (IPP)^30–32^. The formation of IPP is governed by two primary metabolic pathways: the mevalonate (MVA) pathway in the cytosol and the methylerythritol phosphate (MEP) pathway in the plastids^33–35^. Both pathways play important roles in providing precursors not only for rubber biosynthesis but also for other terpenoid-derived metabolites that contribute to plant growth, development, and stress responses^36–38^. Enzymes within these pathways, such as 3-hydroxy-3-methylglutaryl-CoA reductase (HMGR) in the MVA pathway and 1-deoxy-D-xylulose-5-phosphate synthase (DXS) in the MEP pathway, serve as critical regulatory nodes for the two pathways^39–41^. These enzymes are tightly controlled by TFs, including the big six families: AP2/ERF, bHLH, bZIP, MYB, NAC, and WRKY families (Supp. Figure 1), which modulate the expression of these enzymes in response to developmental cues and environmental stresses^42–46^. Investigating the regulation of these pathways, particularly under drought conditions, provides important insights into how guayule evolved its metabolic processes to optimize rubber production while maintaining resilience. This understanding not only enhances our knowledge of guayule’s metabolic flexibility but also highlights opportunities to improve its productivity under challenging environments through genetic and biotechnological approaches.

**Fig. 1:**
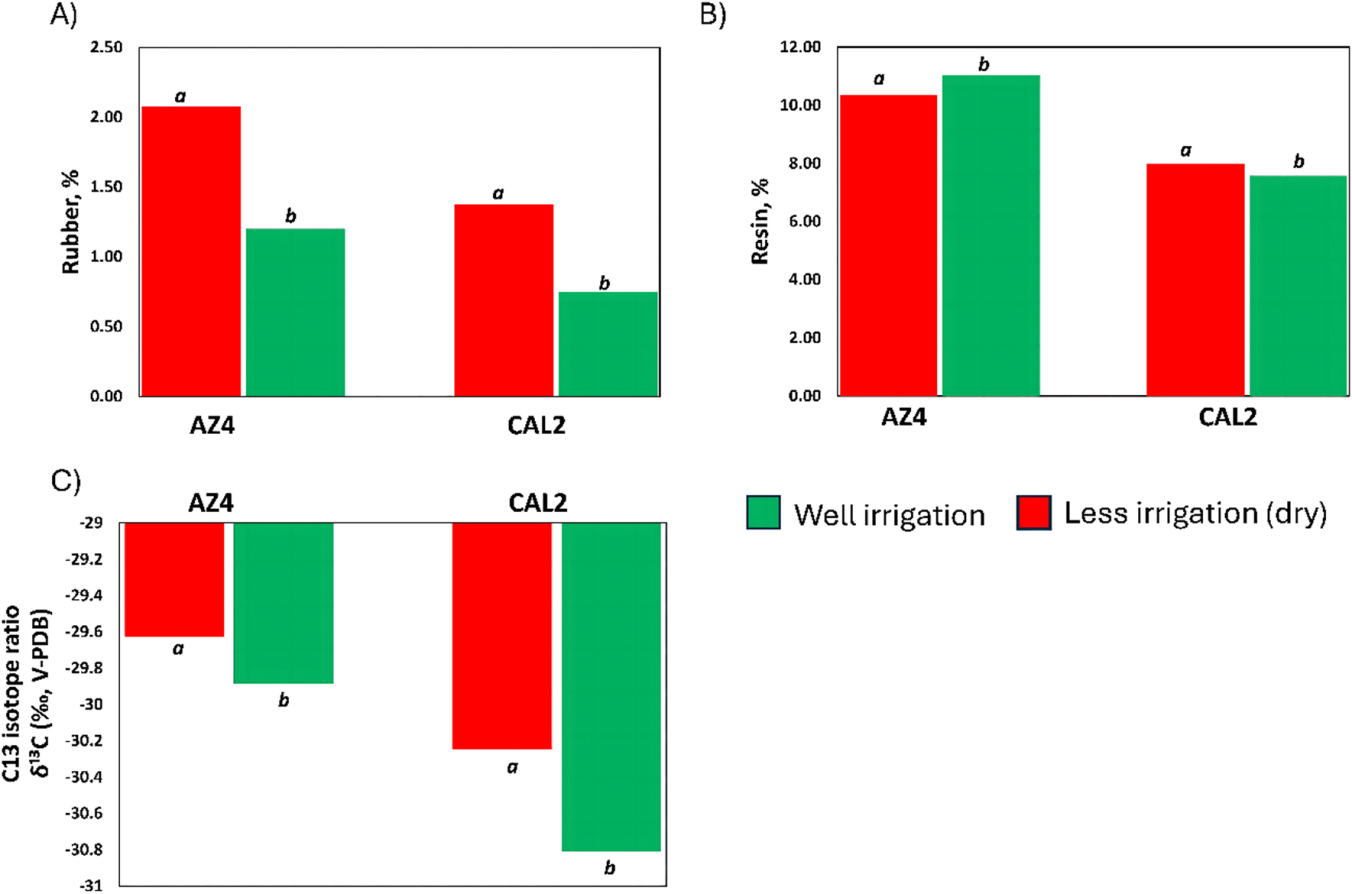
Physiological traits for AZ-4 and CAL-2 plants grown under well irrigated and less irrigated conditions. (**A**) Rubber content (%). (**B**) Resin content (%). (**C**) Water use efficiency (WUE) via δ¹³C (‰, V-PDB). *a*,* b* Significancy level at *p* = 0.05.

Aquaporins are membrane proteins that belong to the major intrinsic protein (MIP) family, with members found in nearly all living organisms^47^. Aquaporins facilitate the transport of water and/or small neutral solutes (urea, boric acid, silicic acid) or gases (ammonia, carbon dioxide)^48^. Based on sequence homology, plant aquaporins are classified into four major subfamilies: the plasma membrane intrinsic proteins (PIP), the tonoplast intrinsic proteins (TIP), the nodulin- 26–like intrinsic membrane proteins (NIPs), and the small basic intrinsic proteins (SIPs)^48,49^. The role of aquaporins in response to abiotic and biotic stresses could be described via regulating plant-water relationships and mechanisms including root hydraulic conductivity, stomatal control and cytoplasmic homeostasis^48,50^. Several studies have shown that expression levels of aquaporin genes vary based on plant species, plant organ, stress type, and time. For example a *TaTIP2* gene was down regulated in response to drought and salt stress exposure of wheat plants^51^, while the *AtPIP2* isoform was up-regulated during both heat and cold treatments of Arabidopsis plants^52,53^. Aquaporins have been widely documented in rubber tree *Hevea brasiliensis*^54,55^. In guayule, aquaporin orthologous PIP1and PIP2 were upregulated in response to drought stress^5,56^.

The current study’s objectives are to: (1) elucidate the drought stress tolerance mechanisms in two guayule cultivars developed for distinct growing regions with different pedigrees, and (2) enable a deeper understanding of its drought-adaptive strategies and potential for sustainable rubber production. By investigating the genetic and regulatory mechanisms governing guayule’s response to water stress, this research not only advances the rubber industry but also contributes to agricultural sustainability in arid regions.

## Materials and methods

### Plant material, growth and drought treatment conditions, and tissue collection

To conduct the transcriptome study, two guayule cultivars were selected: AZ-4 (PI599677; a selection from *P. argentatum* × *P. argentatum*) and CAL-2 (PI478667; a selection from *P. argentatum* × *Parthenium* sp.) based on their contrast responses to less irrigation conditions^10^. AZ-4 and CAL-2, are tetraploids, were developed for Arizona growing conditions Ray, et al.^57^, and California growing conditions Tysdal, et al.^58^ respectively. For each cultivar, one-year transplanted plants were selected from three replicated plots planted in two trials (well-irrigated and low-irrigated) at Maricopa Agricultural Center, University of Arizona, Maricopa AZ (33°03’55’’ N, 111°58’31’’ W). The soil series and textures at the trials were classified as Casa Grande series (fine-loamy, mixed, hyperthermic Typic Natragrids). Water-stressed and well-watered trials were irrigated differently to each suitable stress levels. In general, plots in both well-irrigated and water-stressed trials were furrow irrigated bi-weekly until plants were established, before tissue collection, irrigation was withheld in the water-stressed trial for three months, while well-irrigated trial was irrigated every 2–3 weeks based on weather conditions.

The bark tissues from upper third part of the stem were collected from the three plots representing biological replicates from well-irrigated (IR) and water-stressed (D), trials for each cultivar. Tissues were immediately immersed in liquid N2 and stored at − 80 °C for downstream experiments. Resin and rubber content were determined from said ground samples using a DA7250 At-line NIR Analyzer^59^. Finely powdered samples (approx. 3 mg) were encapsulated in tin and sent to the University of California, Davis for isotope analysis. The 13 C/12 C ratio was expressed relative to the Vienna PeeDee Belemnite (V‑PDB) standard due to the difficulty of measuring absolute isotope composition^60^. Physiological data for these traits were analyzed using analysis of variance (ANOVA), and treatment means were compared with the Least Significant Difference (LSD) test. The LSD procedure was selected because it provides a statistical threshold to determine whether differences among treatments are significant, rather than simply reporting variability, and significance was assessed at the *P* < 0.05 level.

### RNA isolation, cDNA library preparation and sequencing

Total RNA were extracted from the bark tissues using column-based extraction kit, NEBNext Ultra II RNA Library Prep kit (Illumina, San Diego, CA, USA), following the kit’s manufacturer protocol recommendations. The total RNA quantity and purity were estimated using Nanodrop spectrophotometer (Thermo Fisher Scientific Inc., Waltham, MA, USA) and the RNA integrity were assayed using Agilent 2100 Bioanalyzer (Agilent Technologies, Santa Clara, CA, USA). Messenger RNA was purified from total RNA using poly-T oligo-attached magnetic beads. After fragmentation, the first strand of cDNA was synthesized using random hexamer primers, followed by the second strand of cDNA synthesis using mentioned kit. Libraries were quantified using Qubit fluorometer (Thermo Fisher Scientific Inc., Waltham, MA, USA) and size determined using Agilent 2100 Bioanalyzer (Agilent Technologies, Santa Clara, CA, USA). Quantified libraries were pooled and sequenced using NovaSeq 6000 (PE150) platform at Novogene (Sacramento, CA, USA; https://www.novogene.com/us-en/services/research-services/).

### Genome assembly and functional annotation

Following the Novogene’s pipeline, original image data from high-throughput sequencing were transformed into sequenced reads (Raw Data or Raw Reads) by CASAVA base recognition (base calling). Raw data were stored in FASTQ format files, containing sequences and corresponding base quality. Base quality scores were calculated using the Phred score: $$ Qphred = -10log10(e), with “e” represents sequencing error rate, “Qphred” represents base quality values of Illumina platforms. Sequenced raw reads often included low-quality sequences or adapter contamination that could compromise downstream analyses. To ensure data quality, raw reads were filtered to obtain clean reads by: (1) removing reads containing adapter sequences, (2) discarding reads with more than 10% uncertain nucleotides (*N* > 10%), and (3) eliminating reads where over 50% of bases had a quality score below 5. Then reads were trimmed to remove adapter and poly-N sequences (*N* > 10%) and low-quality reads (more than 50% of reads). The GC content of the clean data was calculated. Trinity platform (https://trinityrnaseq.github.io) was used to assemble guayule transcriptome. Hierarchical clustering analysis was done via CORSET software (https://github.com/Oshlack/Corset/wiki) to remove redundance from Trinity, then BU SCO software (https://gitlab.com/ezlab/busco/) was used to evaluate the integrity of the transcripts and provide quantitative assessment of the expected gene content of assembled transcriptome. Gene prediction algorithms were used in conjunction with RNA-seq data to enhance annotation accuracy. Functional annotation involved assigning biological functions to predicted genes using homology-based approaches and compare sequences against known protein databases: NR (NCBI Non-Redundant Protein Sequences; 1E-5 threshold), NT (NCBI Nucleotide Sequences; 1E-5 threshold), PFAM (Protein Family; 1E-2 threshold), KO [KEGG (Kyoto Encyclopedia of Genes and Genome^61–63^ Ortholog; 1E-6 threshold], Swiss-Prot (1E-5 threshold), GO (Gene Ontology; 1E-6 threshold), and KOG (euKaryotic Orthologous Groups; 1E-5 threshold). The iTAK program ^64^(https://itak.feilab.net/cgi-bin/itak/index.cgi) was used to perform the transcription factor analysis of transcripts.

### Differentially expressed transcript analysis, GO and KEGG pathway enrichment

RSEM (version 1.2.28) was used as the quantification tool and not as an aligner. Internally, RSEM relies on Bowtie2 for aligning reads to the Corset-filtered transcriptome, which served as the reference for quantification. FeatureCounts and HTSeq were not applied in this analysis. The raw count data generated by RSEM were used as input for differential expression analysis, ensuring compatibility with downstream statistical tools. DESeq2 (version 1.26.0) was applied exclusively for differential expression analysis, as all sample groups included biological replicates. DESeq2’s statistical framework, which employs negative binomial modeling and shrinkage estimation, is well-suited for handling replicated RNA-seq data. Although edgeR (version 3.28.0) was mentioned in the pipeline documentation, it was not applied to this dataset; its inclusion served only as a contingency for cases without replicates. This approach ensured a consistent and statistically rigorous analysis across all sample groups. The DESeq2 design formula used was ~ treatment, with each pairwise comparison performed independently to evaluate gene expression differences between specific conditions. These included drought versus irrigated treatments within cultivars (e.g., AZ_D vs. AZ_IR, CAL_D vs. CAL_IR), and comparisons across cultivars under the same treatment (e.g., AZ_D vs. CAL_D, AZ_IR vs. CAL_IR). Each group included three biological replicates, providing sufficient statistical power for dispersion estimation. Replicates were balanced across conditions, and principal component analysis (PCA) of normalized counts confirmed that biological replicates clustered tightly within each treatment group, supporting consistency and reproducibility of the dataset. Differential expression was assessed using a false discovery rate (FDR) cutoff of 0.05 and a log₂ fold change threshold of ± 1. Additionally, log₂ fold change shrinkage was applied using DESeq2’s *lfcShrink* function to improve interpretability and reduce the influence of low-count or highly variable genes, particularly for visualization and ranking purposes. FPKM and TPM values derived from RSEM were used exclusively for visualization and descriptive purposes, such as in heatmaps or expression profiles, and were not involved in any statistical testing. Transcripts with adjusted p-values (false discovery rate, FDR, calculated from Benjamini and Hochberg’s approach) below 0.05 and fold changes exceeding a predefined threshold were considered significantly differentially expressed. GO enrichment analysis was performed to categorize differentially expressed genes (DEGs) into three main domains: biological processes (BP), molecular functions (MF), and cellular components (CC). Statistical methods, such as Fisher’s exact test or hypergeometric distribution, were applied to identify significantly enriched GO terms. Kyoto Encyclopedia of Genes and Genomes (KEGG) pathway enrichment analysis was conducted to map DEGs to metabolic and signaling pathways. Enrichment scores were calculated to identify pathways significantly associated with the experimental conditions. The upregulation or downregulation of GO and KEGG pathways was determined through pathway enrichment analysis of differentially expressed transcripts, conducted using Novogene’s standard bioinformatics pipeline. This approach identifies statistically significant pathways based on the direction and magnitude of gene expression changes. For example, when a majority of genes associated with a pathway are downregulated in a given condition, the pathway is classified as downregulated. The analysis utilized the KEGG Automatic Annotation Server (KAAS) to perform pathway mapping and statistical testing.

### BLAST-based exploration of annotated transcription factors and aquaporins

TF and aquaporin identification was performed using both domain-based prediction and sequence similarity approaches. For TF prediction, the pipeline employed iTAK, which uses *hmmscan* to identify transcription factors based on predefined TF family models and classification rules, as described previously^65,66^. This enabled the identification of homologous sequences belonging to key TF families, including the three bidirectionally regulated under drought (AP2/ERF, MYB, NAC) and the six major TF families (AP2/ERF, bHLH, bZIP, MYB, NAC, WRKY) associated with the MVA and MEP pathways. For aquaporin identification and broader functional annotation, BLAST-based searches were conducted against multiple curated databases including NR, SwissProt, and KEGG, using Diamond and NCBI BLAST with an e-value threshold of ≤ 1e-5, retaining the top 10 alignment hits per unigene. Additional domain-based annotation was performed using Pfam via HMMER (*hmmscan*) with an e-value threshold of 0.01, and GO annotation was derived using Blast2GO with an e-value threshold of 1e-6. This process enabled identification of aquaporin homologs across the four major plant subfamilies: PIP, TIP, NIP, and SIP. To collapse isoforms and reduce redundancy, Corset was applied prior to annotation. Corset clusters transcripts based on shared read support and expression profiles, effectively grouping isoforms into representative clusters that approximate gene-level resolution. All TF and aquaporin counts reported in the study reflect these non-redundant gene-level assignments, derived from Corset-filtered transcript clusters and annotated using the thresholds and tools described above. This ensures that the reported numbers are not inflated by isoform redundancy and are consistent with gene-level interpretation.

## Result

### Effects of droughts stress on rubber, resin and water use efficiency of two guayule cultivars

To gain deeper understanding of responses of AZ-4 and CAL-2 to drought stress conditions, rubber content and resin content were estimated. Data revealed AZ-4 and CAL-2 are significantly different in their rubber, as well as their interaction to drought stress conditions (Fig. 1). AZ-4 significantly produced more rubber than CAL-2 (2.1% vs. 1.4% under drought; 1.2% vs. 0.7% under well-irrigated conditions; Fig. 1A). in both cultivars, rubber content was increased significantly when plants were grown under drought stress conditions. Indicating AZ-4 and CAL-2 have different mechanisms to cope with drought stress. Even though have significant differences in resin content, where AZ-4 was higher than CAL-2 (10.2% vs. 8.1% under drought; 11.0% vs. 7.8% under well-irrigated conditions; Fig. 1B), resin content was not affected by drought stress conditions. As well, data should that AZ-4 resists drought stress conditions though increase water use efficiency (WUE) (increased δ¹³C isotope discrimination ratio) compared to CAL2 under same conditions (Fig. 1C). Isotopic analysis showed that AZ-4 had δ¹³C values of − 29.6 under drought and − 29.9 under well-irrigated conditions, compared to − 30.2 and − 30.8, respectively, for CAL-2 (Fig. 1C). Together, these results indicate that AZ-4 exhibits higher rubber and resin contents, and potentially greater water use under drought compared to CAL-2. These contrasting physiological traits make both cultivars valuable candidates for transcriptomic studies of guayule under drought stress.

### Transcriptome sequencing and read assembly

To explore molecular responses tied to drought stress tolerance mechanisms and potential rubber biosynthesis pathways in guayule cultivars, AZ-4 and CAL-2, twelve RNA libraries from plants grown under drought stress and irrigated stem tissues were sequenced using the NovaSeq 6000 platform. This generated 457,077,694 raw pair-end reads, with 98.75% of cleaned reads (451,344,416) retained for assembly. Clean reads were assembled using the Trinity platform following the verified pipeline described in Sect.  2.3, with redundant transcripts removed by CORSET and transcriptome completeness assessed by BUSCO to ensure a high-quality and comprehensive assembly. This yielded 440,984 unique transcripts (non-redundant at 95%), with transcripts > 300 bp retained. Expressed transcripts (99.4% of normalized data) achieved an N50 of 1361 bp, totaling 456.7 Mb in size, with lengths ranging from 301 to 15,761 bp. This means that 50% of the total transcriptome assembly length is contained in transcript sequences that are at least 1361 base pairs long, suggesting a reasonable level of assembly continuity. Additionally, 72.96% of clean reads mapped back to reference transcriptomes (e.g., *Helianthus annuus* L., *Ambrosia artemisiifolia* L., *Artemisia annua* L.) (detailed mapping statistics per sample are included in Supp. Table 1. Detailed read lengths and layout are in Supp. Figure 2).

**Table 1:** Identification and expression analysis of aquaporin transcripts under drought and well-irrigated conditions for AZ-4 and CAL-2. Am: *Antirrhinum majus*; At: *Arabidopsis thaliana*; Ha: *Helianthus annuus*; Ht: *Helianthus tuberosus*; Ma: *Musa acuminata*; Os: *Oryza sativa*; Zm: *Zea mays.* Highlighted: overlapped clusters between two data sets.

Gene ID	Log2 FoldChange	Adjusted *p*-value	Gene Length	Family	Homolog(s)
**AZ-4_Drought vs. AZ-4_Irrigated**					
Cluster-32812.63634	-4.1344	8.08E-21	2593	NIP	HaNIP6-1 AtNIP6-1
Cluster-32812.56264	-1.8603	0.042482	648	NIP	HaNIP5-1
Cluster-32812.79978	-1.6762	3.73E-05	936	NIP	HtNIP1-1B ZmNIP2-2
Cluster-32812.48395	-3.3688	5.54E-06	341	PIP	HaPIP2-4 AtPIP2-5
Cluster-32812.48382	-2.7076	4.74E-09	1040	PIP	HaPIP2-4 AtPIP2-4
Cluster-32812.44828	-2.5812	0.000238	329	PIP	HaPIP2-4
Cluster-32812.56239	-1.9256	5.03E-11	1074	PIP	HaPIP2-5 AtPIP2-5
Cluster-32812.48396	-1.8902	3.66E-07	395	PIP	HaPIP2-4 AtPIP2-5
Cluster-32812.60120	-1.8283	0.001879	1230	PIP	HtPIP2-2 HtPIP2-5
Cluster-32812.48766	-1.6716	0.000286	416	PIP	HaPIP2-5 AtPIP2-5
Cluster-32812.48398	-1.3057	0.000118	979	PIP	HaPIP2-4 ZmPIP2-1
Cluster-32812.55611	-1.3018	0.014136	761	PIP	HaPIP2-5 ZmPIP2-4
Cluster-32812.59788	-1.1209	0.000663	1391	PIP	HaPIP2-7 AtPIP2-7
Cluster-32812.40945	2.4751	0.021781	1347	PIP	HaPIP1-4
Cluster-32812.95244	7.3948	0.000234	484	PIP	MaPIP1-2
Cluster-32812.38396	-2.2691	3.00E-09	1469	SIP	HaSIP2-1 AtSIP2-1
Cluster-45262.0	-6.5507	0.044042	511	TIP	HaTIP-type AmTIP-type
Cluster-32812.55497	-4.1461	7.27E-05	1870	TIP	HaTIP4-1 AtTIP4-1
Cluster-32812.9051	-3.3614	3.38E-06	1021	TIP	HaTIP1-3 MaTIP1-2
Cluster-32812.55617	-2.5693	7.09E-25	1005	TIP	HaTIP2-1 AtTIP2-1
Cluster-32812.48194	-1.9577	4.34E-08	1111	TIP	AtTIP2-1
Cluster-32812.54678	-1.8196	5.93E-13	1328	TIP	HaTIP2-1 AtTIP2-1
Cluster-32812.48193	-1.6339	0.004659	351	TIP	HaTIP2-1 AtTIP2-1
**CAL-2_Drought vs. CAL-2_Irrigated**					
Cluster-32812.63634	-1.6457	4.46E-05	2593	NIP	HaNIP6-1 AtNIP6-1
Cluster-32812.55620	1.3222	2.32E-05	1193	NIP	HaNIP5-1 AtNIP5-1
Cluster-32812.50731	1.676	3.38E-06	838	NIP	HaNIP5-1 OsNIP3-1
Cluster-32812.64459	2.1881	2.34E-11	1379	NIP	HaNIP5-1 OsNIP3-1
Cluster-32812.48395	-3.6736	3.59E-10	341	PIP	HaPIP2-4 AtPIP2-5
Cluster-32812.69020	-3.6432	0.002139	343	PIP	HaPIP2-4
Cluster-32812.48393	-3.0399	0.00058	410	PIP	HtPIP2-2 AtPIP2-5
Cluster-32812.48382	-2.8146	2.47E-08	1040	PIP	HaPIP2-4 AtPIP2-4
Cluster-32812.44828	-2.7311	0.007482	329	PIP	HaPIP2-4
Cluster-32812.48766	-2.0558	3.78E-06	416	PIP	HaPIP2-5 AtPIP2-4
Cluster-32812.55611	-2.0204	0.001505	761	PIP	HaPIP2-5 ZmPIP2-4
Cluster-32812.56239	-1.6995	7.63E-08	1074	PIP	HaPIP2-5 AtPIP2-5
Cluster-32812.59788	-1.6687	1.63E-09	1391	PIP	HaPIP2-7 AtPIP2-7
Cluster-32812.46618	-1.4913	7.24E-06	546	PIP	HaPIP1-3
Cluster-32812.48396	-1.4657	0.029357	395	PIP	HaPIP2-4 AtPIP2-5
Cluster-32812.52520	1.1961	0.003463	1023	PIP	HaPIP1-3 AtPIP1-2
Cluster-32812.38294	1.2655	0.047454	1026	PIP	HtPIP2-8 AtPIP2-8
Cluster-32812.23626	1.5012	1.75E-05	1327	PIP	HaPIP2-5 ZmPIP2-5
Cluster-32812.66556	-1.1128	7.96E-06	1015	SIP	HaSIP1-1 OsSIP1-1
Cluster-32812.9051	-4.242	2.90E-09	1021	TIP	HaTIP1-2 MaTIP1-2
Cluster-32812.55617	-3.284	1.55E-45	1005	TIP	HaTIP2-1 AtTIP2-1
Cluster-32812.55497	-2.7616	2.28E-33	1870	TIP	HaTIP4-1 AtTIP4-1
Cluster-32812.48194	-2.3408	1.91E-21	1111	TIP	AtTIP2-1
Cluster-32812.54678	-2.2211	4.07E-17	1328	TIP	HaTIP2-1 AtTIP2-1
Cluster-32812.48193	-1.7469	0.000175	351	TIP	HaTIP2-1 AtTIP2-1
Cluster-29402.0	8.1708	2.45E-05	1096	TIP	TIP-type

**Fig. 2:**
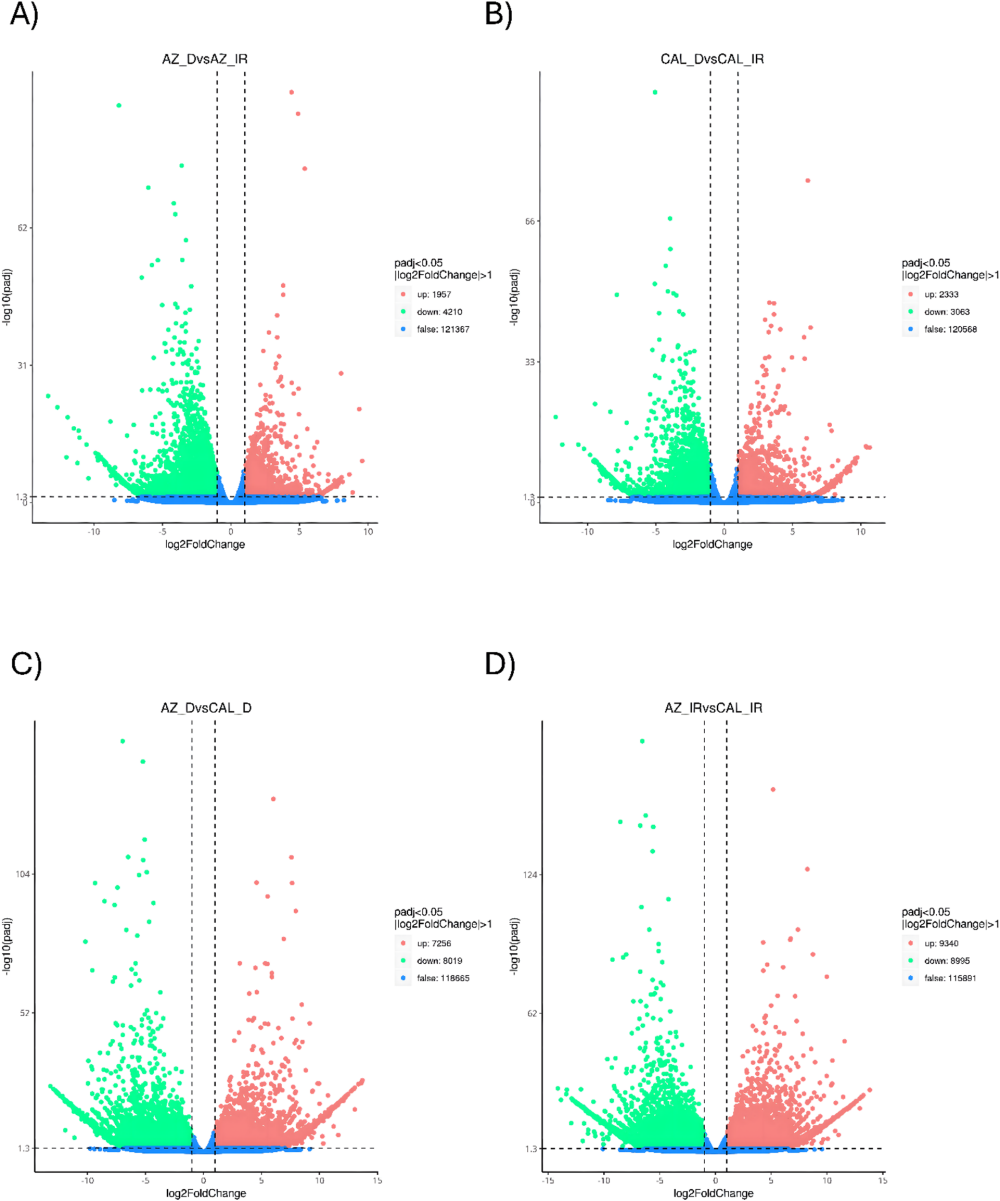
Differentially expressed transcripts in guayule accessions under drought conditions. (**A**) Differentially expressed transcripts under drought and irrigated conditions in AZ-4. (**B**) Differentially expressed transcripts under drought and irrigated conditions in CAL-2. (**C**) Differentially expressed transcripts under drought in AZ-4 and CAL-2. (**D**) Differentially expressed transcripts under drought versus irrigated conditions in guayule (both cultivars combined). Green: Significantly downregulated; Red: Significantly upregulated.

### Differential gene expression and functional enrichment

To quantify unigene abundances, FPKM values were calculated and differentially expressed transcripts were identified by RSEM with further run via DESeq2 (FDR < 0.05, log2 fold change > 1). For AZ-4 plants grown under drought stress and irrigated conditions, 6167 transcripts were significantly differentially expressed (4210 down, 1957 up; Fig. 2A). CAL-2 had 5396 transcripts (3063 down, 2333 up; Fig. 2B). Comparing AZ-4 and CAL-2 plants grown under drought conditions, 15,275 transcripts were differentially expressed (8019 down, 7256 up; Fig. 2C). Across drought and irrigated conditions, 5350 transcripts were significantly expressed (3474 up, 1876 down; Fig. 2D).

To achieve comprehensive gene functional annotation, seven databases were used. The function and characteristics of these databases include NR (NCBI Non-Redundant Protein Sequences) with 75,357 transcripts identified (50.39%), NT (NCBI Nucleotide Sequences) with 67,245 transcripts identified (44.96%), PFAM (Protein Family) with 45,517 transcripts identified (30.43%), KO [KEGG (Kyoto Encyclopedia of Genes and Genome) Ortholog] with 22,952 transcripts identified (15.34%), Swiss-Prot with 46,747 transcripts identified (31.26%), GO (Gene Ontology) with 44,887 transcripts identified (30.01%), and KOG (euKaryotic Orthologous Groups) with 11,201 transcripts identified (7.49%).

**Fig. 3:**
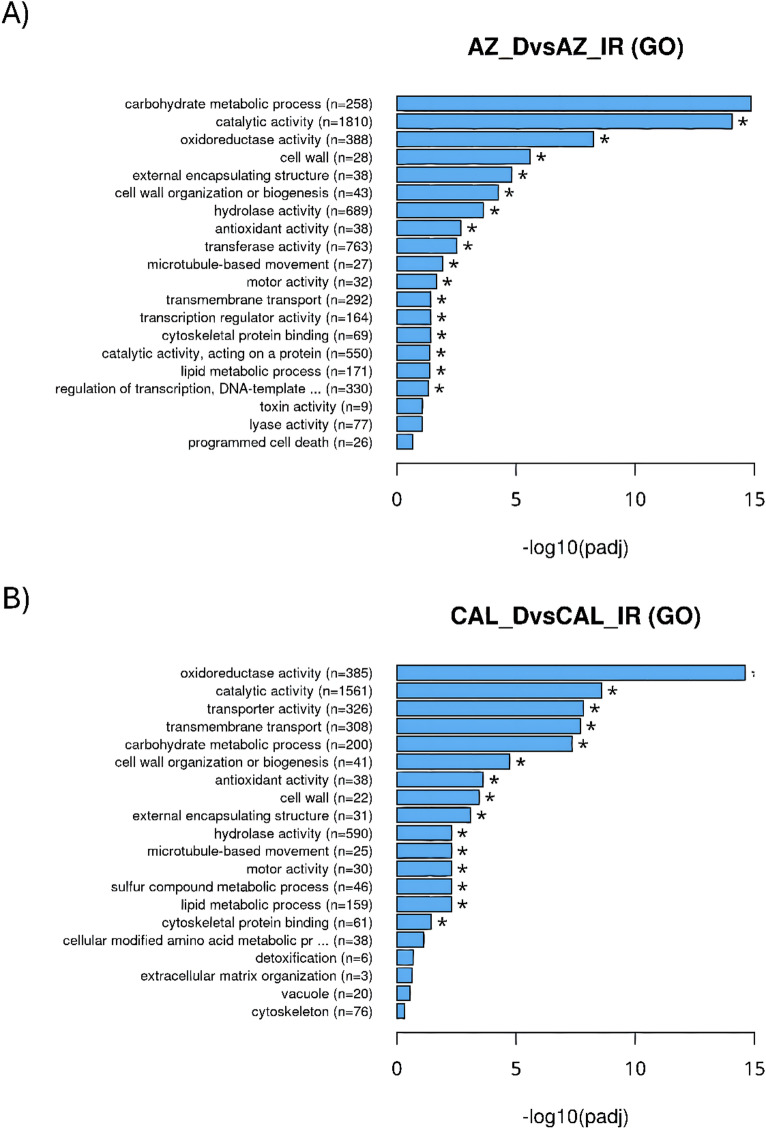
Functional classification and pathway analysis of guayule transcripts via GO. GO assignments classify transcript functions into biological process, molecular function, and cellular component categories for AZ-4 (**A**) and CAL-2 (**B**) under drought and irrigated conditions.

The GO analyses identified potential functions of sequenced transcripts, classifying them into biological process, molecular function, and cellular component categories for AZ-4 and CAL-2 plants grown under drought stress and well irrigated conditions. In AZ-4, 36,351 contigs were assigned across 121 sub-categories, with the most significant categories were being ‘carbohydrate metabolic process’ [− log10(padj) = 14.85], ‘catalytic activity’ (14.05), and ‘oxidoreductase activity’ (8.24) (Fig. 3A). Similarly, in CAL-2, 36,351 contigs were assigned to 119 sub-categories, with significant functions including ‘oxidoreductase activity’ [− log10(padj) = 14.61], ‘catalytic activity’ (8.58), and ‘transporter activity’ (7.83) (Fig. 3B). GO term categories with lower significant adjusted p-values are listed in Supp. Table 2. Three sub-categories overlapped between the two cultivars, including ‘catalytic activity,’ which accelerates biochemical reactions for efficient resource use, and ‘oxidoreductase activity,’ that are important for ROS management and oxidative stress mitigation. The two GO terms ‘cyclase activity’ and ‘histone binding’ were exclusively identified in AZ-4, while the GO term ‘nitrogen cycle metabolic process’ was unique to CAL-2, though these terms did not meet the adjusted p-values cutoff.

**Fig. 4:**
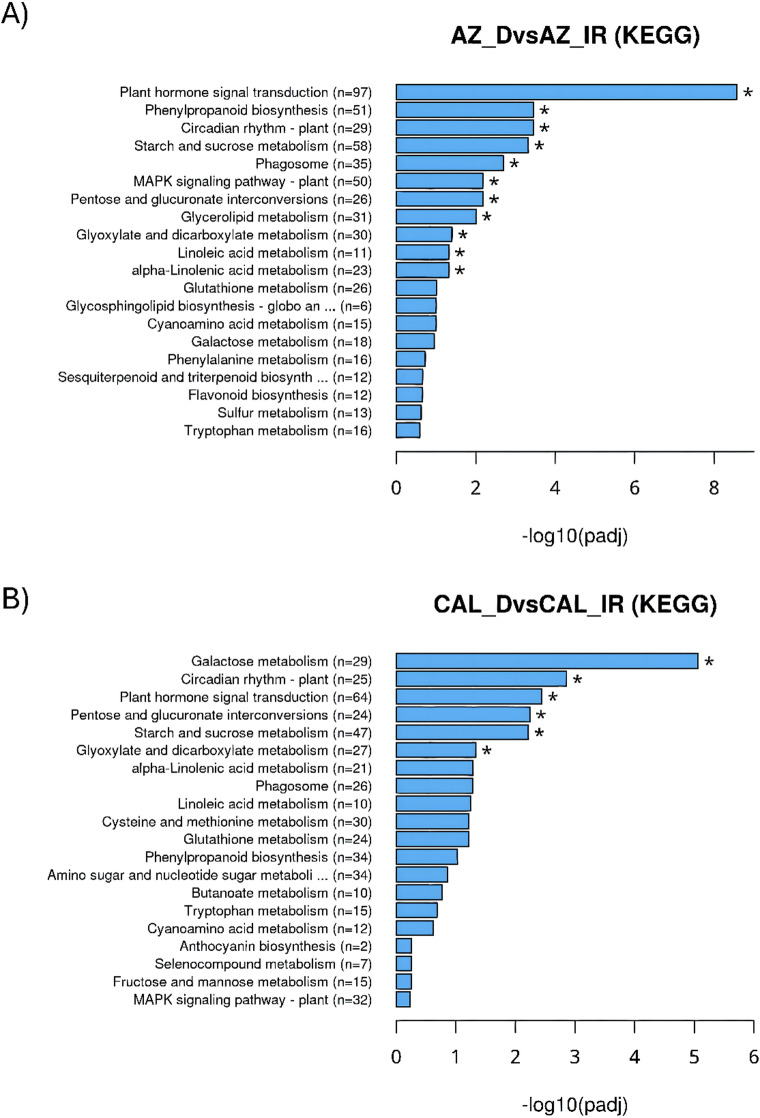
Functional classification and pathway analysis of guayule transcripts via KEGG. KEGG pathway analysis identified key biological pathways from annotated transcript sequences in AZ-4 (**A**) and CAL-2 (**B** under drought and irrigated conditions.

KEGG pathway analysis identified active biological pathways in annotated transcript sequences from AZ-4 and CAL-2 under drought and well irrigated conditions. In AZ-4, 13,652 transcripts were assigned to 115 metabolic pathways, with the most significant pathways were ‘plant hormone signal transduction’ [− log10(padj) = 8.57], ‘circadian rhythm – plant’ (3.45), ‘phenylpropanoid biosynthesis’ (3.45), ‘starch and sucrose metabolism’ (3.32), and ‘phagosome’ (2.69) (Fig. 4A). Similarly, in CAL-2, 13,652 transcripts were assigned to 121 pathways, with significant ones including ‘galactose metabolism’ [− log10(padj) = 5.06], ‘circadian rhythm – plant’ (2.85), ‘plant hormone signal transduction’ (2.44), ‘pentose and glucuronate interconversions’ (2.24), and ‘starch and sucrose metabolism’ (2.22) (Fig. 4B). KEGG pathways with lower significant adjusted p-values are listed in Supp. Table 3. Among highly significant pathways, three were shared between the cultivars: ‘plant hormone signal transduction,’ ‘circadian rhythm – plant,’ and ‘starch and sucrose metabolism.’

### Upstream regulation of drought response analysis

**Fig. 5:**
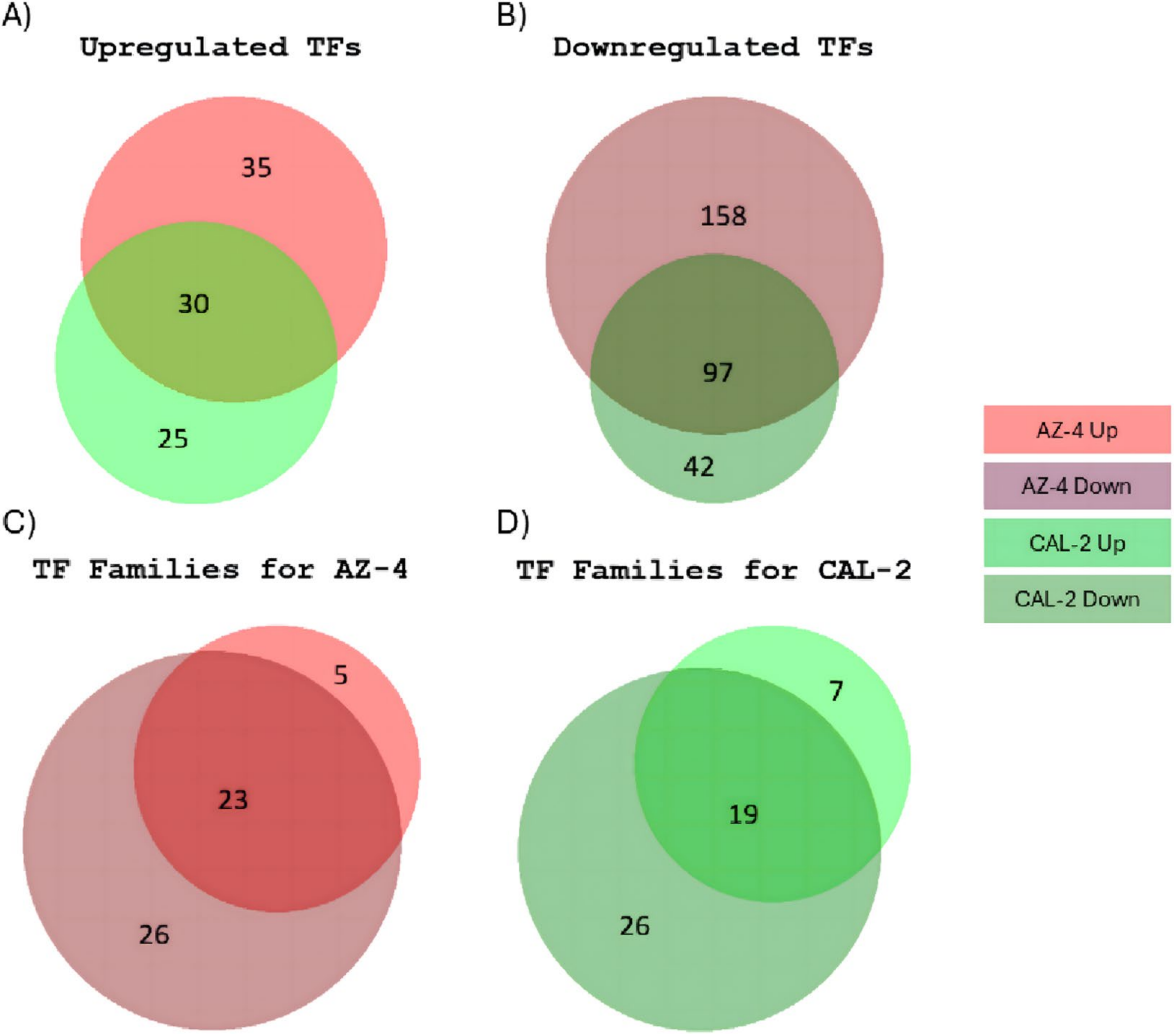
Comparison of upregulated and downregulated TFs in guayule cultivars. (**A**) Overlapped TFs in upregulated transcripts between AZ-4 and CAL-2 under drought stress conditions. (**B**) Overlapped TFs in downregulated transcripts between AZ-4 and CAL-2 under drought stress conditions. (**C**) TF family analysis for AZ-4. (**D**) TF family analysis for CAL-2. Green: CAL-2; Red: AZ-4; Lighter shade: upregulated; Darker shade: downregulated.

Transcription factors (TFs) are key regulators of gene transcription, impacting plant growth, development, and stress responses. To understand how guayule plants cope with drought, TFs were annotated as a proxy for transcriptional regulatory mechanisms. Our findings identified 2,929 transcripts encoding putative TFs, and were classified into 69 families. The most abundant families include myeloblastosis (MYB) and related (242, 8.26%), AP2/ERF (189, 6.45%), WRKY (137, 4.68%), C2H2 (136, 4.64%), and bHLH (131, 4.47%). Under drought stress and irrigated conditions revealed 1,957 significantly upregulated transcripts were identified in AZ-4, with 1,608 (96.4%) annotated in at least one of seven databases. Filtering identified 65 TFs (3.3% of upregulated transcripts). For CAL-2, 2,333 transcripts were significantly upregulated, of which 1,977 (84.7%) were annotated. Further filtering identified 55 TFs (2.8% of upregulated transcripts). Combining data from both cultivars revealed 30 overlapped TFs (Fig. 5A). Of 4,210 significantly downregulated TF transcripts, 3,940 (93.6%) were annotated. Filtering classified 255 TFs (6.1%) from characterized families. In CAL-2 samples, 3,063 downregulated transcripts were identified, with 2,777 (90.7%) annotated. Further filtering yielded 139 TFs (4.5%) from characterized families. Comparing the two cultivars revealed 97 overlapping downregulated TFs (Fig. 5B).

**Fig. 6:**
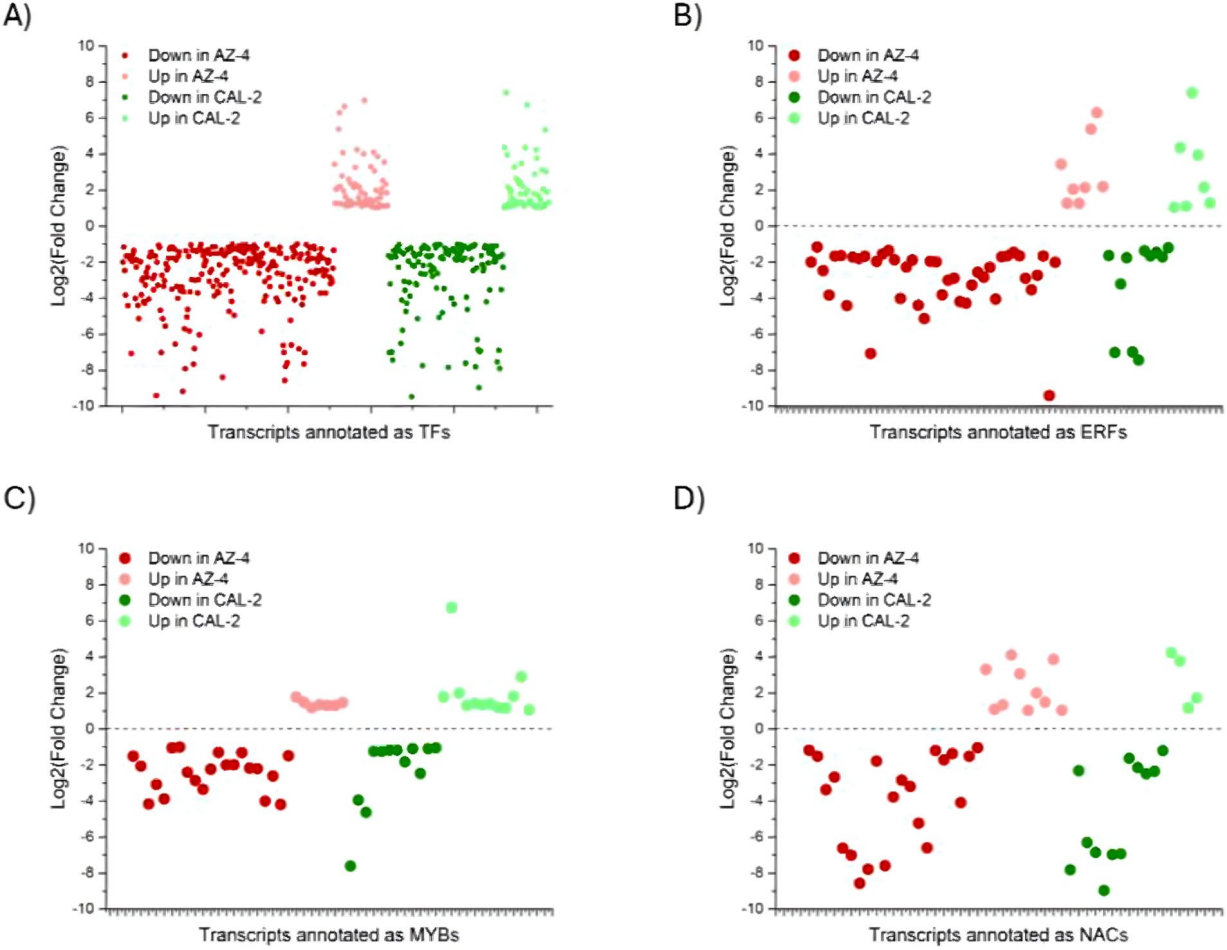
Regulatory dynamics of key TF families in drought-stressed guayule. (**A**) Shared upregulated and downregulated TFs in AZ-4 and CAL-2 under drought. (**B** − **D**) TF families AP2/ERF, MYB, and NAC and their bidirectional regulation under drought conditions in both cultivars. Green: CAL-2; Red: AZ-4; Lighter shade: upregulated; Darker shade: downregulated.

Under drought stress conditions, examining 30 upregulated and 97 downregulated TFs in both cultivars provided insights into guayule’s drought tolerance (Fig. 5A). TFs from AZ-4 were classified into 54 gene families, with 28 found in the upregulated transcripts and 49 in the downregulated, and 23 overlapped families across all transcripts (Fig. 5C). TFs identified from CAL-2 were categorized into 52 gene families, with 26 identified in the upregulated transcripts and 45 gene families in the downregulated transcripts and 19 overlapped families (Fig. 5D). Among the identified gene families, five gene families were exclusively found in the upregulated transcripts of AZ-4 plants, and seven gene families were exclusively found in the upregulated transcripts of CAL-2 plants. In contrast, each cultivar had 26 gene families were exclusively identified in the downregulated transcripts. Among the identified families, AP2/ERF, MYB, and NAC gene families were the top members that were both upregulated and downregulated under drought stress conditions in both cultivars (Fig. 6B − D).

The APETALA2/Ethylene Response Factor (AP2/ERF) family in *Arabidopsis* includes over 100 genes, known for their roles in stress responses. In drought-stressed guayule, homologs of ERF105, DREB2B, DREB2A, DREB2C, ERF1B, ERF34, and ERF40 were upregulated in both cultivars. Nine ERF family members, ERF71, ERF72, ERF5, ERF6, ERF14, ERF22, ERF40, ERF36, and ERF59, were consistently downregulated in both AZ-4 and CAL-2 under drought conditions. Current data revealed that guayule upregulated MYB59, MYB48, LHY1, MYBS5, MYBS2, RVE8, and RVE1 under drought stress conditions. MYB59, MYB48, MYBS5, MYBS2, LHY1, and RVE subfamily members (RVE8 and RVE1) were consistently upregulated in both AZ-4 and CAL-2. Conversely, six MYB members, MYB91, MYB46, MYB24, MYB44, DIV1, and KUA1, were downregulated in both cultivars under drought stress conditions. RNA-Seq analysis showed that both AZ-4 and CAL-2 upregulated homologs of NAC family members including NAC072, NAC102, and NAC019 under drought conditions. Conversely, six NAC family members, NAC03, NAC043, NAC086, NAC083, NAC01, and NAC090, were downregulated in both cultivars.

### TFs that participate in the MVA and MEP pathways under drought-stress conditions

**Fig. 7:**
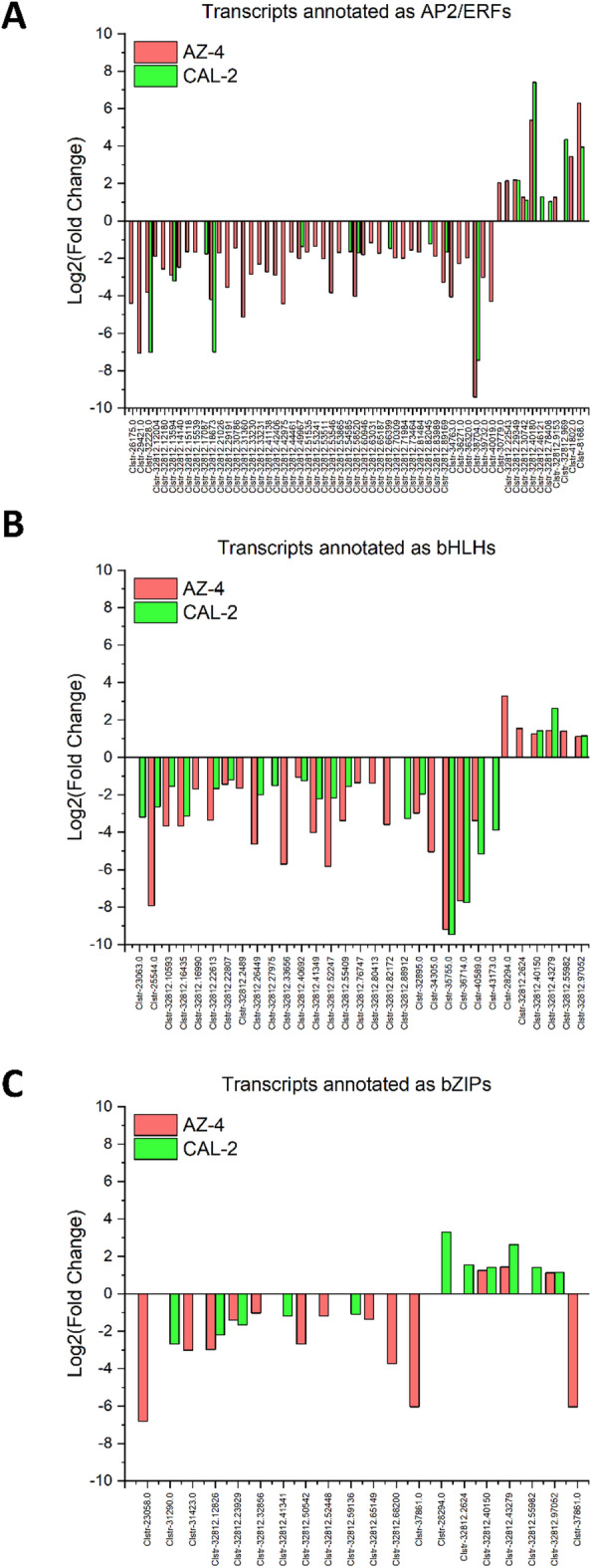
Regulatory dynamics under drought of the AP2/ERF, bHLH, and bZIP families for enzymes in the MVA and MEP pathways. (**A**) Bidirectional regulation of transcripts annotated as AP2/ERFs. (**B**) Bidirectional regulation of transcripts annotated as bHLHs. (**C**) Bidirectional regulation of transcripts annotated as bZIPs. Green: CAL-2; Red: AZ-4.

Rubber biosynthesis in guayule is controlled by isopentenyl pyrophosphate (IPP), mono-, di-, and triterpene, synthesized via the cytosolic mevalonate (MVA) and methylerythritol phosphate (MEP) pathways^30,33,34^. Studies from other model plants have identified multiple TF families (Supp. Figure 1), such as the AP2/ERF family, as important regulators in these pathways^30,33,34^. These TFs work on 3-hydroxy-3-methylglutaryl-CoA reductase (HMGR) enzymes to generate mevalonate, and further downstream on geranylgeranyl pyrophosphate synthase (GGPPS) and farnesyl pyrophosphate synthase (FPPS), precursors of diterpene and triterpene, and even on terpene synthase (TPS) to synthesize diterpene and triterpene in the MVA pathway. For the MEP pathway, AP2/ERF TFs regulate 1-deoxy-D-xylulose-5-phosphate synthase (DXS) and 1-deoxy-D-xylulose-5-phosphate reductoisomerase (DXR) to make MEP from glyceraldehyde-3-phosphate (G3P) and pyruvate, and later work on 4-hydroxy-3-methylbut-2-enyl diphosphate synthase (HDS) to make the compound 4-hydroxy-3-methylbut-2-enyl pyrophosphate (HMBPP), and 4-hydroxy-3-methylbut-2-enyl diphosphate reductase (HDR), isopentenyl diphosphate isomerase (IDI) to make dimethylallyl pyrophosphate (DMAPP) and IPP, and further downstream on GPPS and TPS to synthesize geranyl pyrophosphate (GPP), monoterpene, and diterpene. Under drought stress conditions, AZ-4 had 42 downregulated and 8 upregulated AP2/ERF TFs, while CAL-2 had 11 downregulated and 7 upregulated AP2/ERF TFs. Among downregulated TFs, 7 overlapped, and among upregulated, 4 overlapped (Fig. 7A).

Previous studies also indicated the basic helix-loop-helix (bHLH) family as important regulators in the MVA and MEP pathways^42,43^. It is known that the bHLH family acts throughout both MVA and MEP pathways. In the MVA pathway, bHLH regulates HMGS and HMGR enzymes, prior to the generation of the compound MVAPP, and further downstream enzymes: GGPPS, FPPS, and TPS, for di- and triterpene biosynthesis^67^. Likewise, in the MEP pathway, this family regulates DXS, DXR, 4-diphosphocytidyl-2-C-methyl-D-erythritol kinase (CMK), and HDS to make the compound HMBPP, and later IDI to make DMAPP and IPP, and on GPPS and TPS to make GPP, mono- and diterpene. Under drought stress conditions, 21 and three bHLH TFs were downgraded and upregulated respectively in AZ-4, while 18 downregulated and six upregulated bHLH TFs were identified in CAL-2. Among downregulated bHLH TFs, 14 were identified in both cultivars, while two were identified among upregulated bHLH TFs (Fig. 7B).

The next transcription factor family influencing the MVA and MEP pathways is bZIP, which regulates a broad range of genes encoding enzymes in these two pathways in oilseed crops, such as *Arabidopsis* and *Brassica*^68^, and medicinal plants, such as tea plants and *Bupleurum*^46,69^. For the MVA pathway, the bZIP family acts on HMGS, HMGR, mevalonate kinase (MK), phosphomevalonate kinase (PMK) to produce mevalonate diphosphate (MVAPP), and mevalonate diphosphate decarboxylase (MDD) and IPPI to generate DMAPP and IPP, and TPS to synthesize diterpene and triterpene. Similarly, for the MEP pathway, bZIP regulates 2-C-methyl-D-erythritol 4-phosphate cytidylyltransferase (CMS) and HDR to produce IPP and DMAPP, and further downstream directly on TPS to synthesize mono- and diterpene^70,71^. Hence, by modulating gene activity, bZIP TFs influence the flow of metabolites and affect natural rubber production. In the current study, four upregulated and ten downregulated bZIP TFs were expressed in AZ-4, while CAL-2 had five downregulated TFs and none upregulated. There were two downregulated bZIP TFs identified in both cultivars (Fig. 7C).

**Fig. 8:**
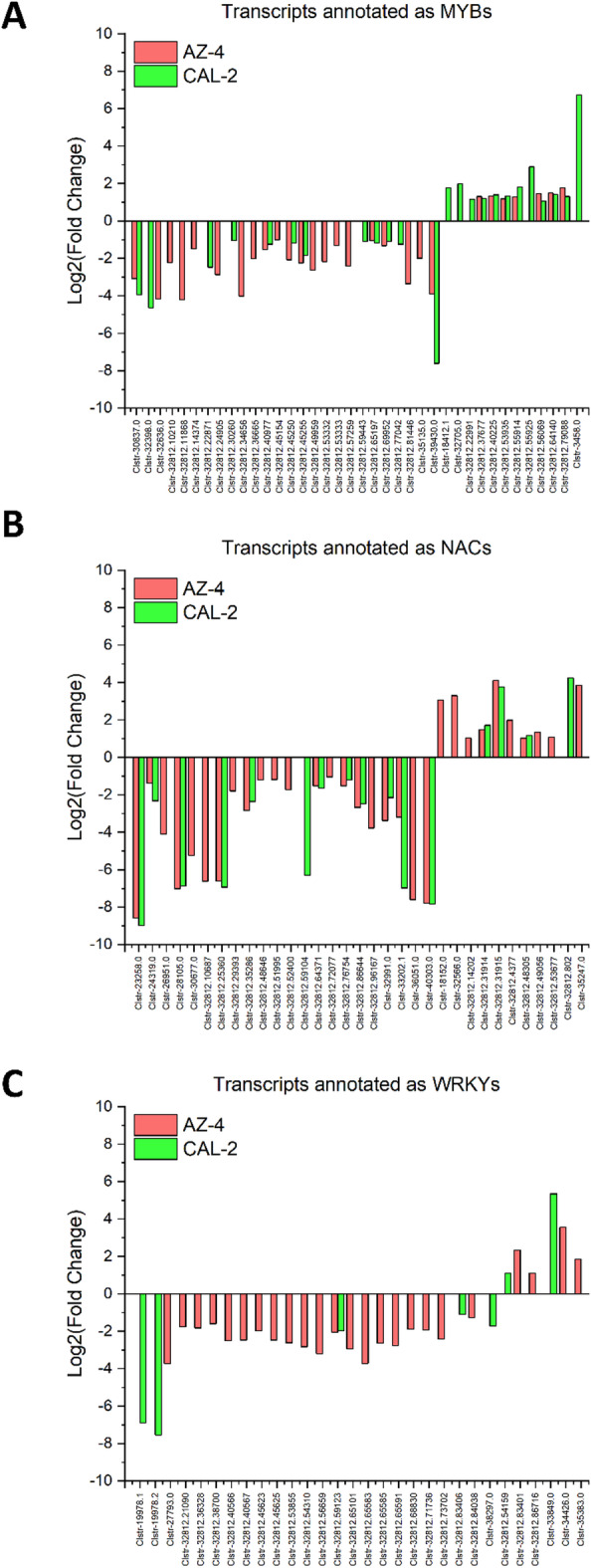
Regulatory dynamics under drought of the NAC, MYB, and WRKY families for enzymes in the MVA and MEP pathways. (**A**) Bidirectional regulation of transcripts annotated as NACs. (**B**) Bidirectional regulation of transcripts annotated as MYBs. (**C**) Bidirectional regulation of transcripts annotated as WRKYs. Green: CAL-2; Red: AZ-4.

The NAC transcription factor family regulates the MVA and MEP pathways, which are important for terpenoid biosynthesis in *Arabidopsis*^72^, medicinal plant *Thymus daenensis*^42^ and tea plants^69^, including those essential for plant growth and defense, such as in oregano^45^. The NAC TF family acts on HMGS, HMGR, MK, and PMK to produce MVAPP, and later on GGPPS and FPPS to generate GGPP and FPP, precursors of diterpene and triterpene, respectively, in the MVA pathway. Likewise, in the MEP pathway, the NAC family regulates CMK to assist in the generation of HMBPP, works on HDR to synthesize DMAPP and IPP, and later regulates GPPS to produce GPP and GGPP, precursors of mono- and diterpene. NAC TFs also support drought stress responses by regulating genes involved in stomatal activity, osmotic balance, and reactive oxygen species (ROS) detoxification^73^. Under drought stress conditions, 21 downregulated and 10 upregulated NAC TFs were expressed in AZ-4, while in CAL-2 plants, 12 downregulated and four upregulated TFs were expressed. In both cultivars, 11 downregulated NAC TFs were overlapped, and three upregulated TFs overlapped (Fig. 8A).

The MYB transcription factor family is well-documented as a key regulator of natural rubber biosynthesis. Members of this family regulate acetoacetyl-CoA thiolase (AACT), 3-hydroxy-3-methylglutaryl-CoA synthase (HMGS), and HMGR enzymes, which are responsible for the early steps of fixing acetyl-CoA and synthesizing mevalonate in the MVA pathway^73^. Similarly, in the MEP pathway, MYB TFs work on DXS, DXR, CMK, and HDS to fix G3P and pyruvate and generate HMBPP, as well as HDR and IDI to synthesize DMAPP and IPP. In the current study, seven upregulated and 21 downregulated MYB TFs were expressed in AZ-4, while an equal number of 12 upregulated and 12 downregulated MYB TFs expressed in CAL-2. There were 14 downregulated MYB TFs were expressed in both cultivars, and all 7 upregulated MYB TFs for AZ-4 were also part of the 12 upregulated MYB TFs for CAL-2 (Fig. 8B).

Another transcription factor family regulating the MVA and MEP pathways is WRKY^62,67^, which modulates genes involved in metabolic and stress-response pathways, including terpenoids. This family works on HMGR to help generate mevalonate and later MVAPP, and further downstream on GGPPS and FPPS to produce GGPP and FPP, precursors of diterpene and triterpene in the MVA pathway^67^. Similarly, WRKY TFs regulate DXS and DXR to initiate the MEP pathway from G3P and pyruvate, and later IDI to generate DMAPP and IPP, and even further downstream on GGPPS and FPPS to synthesize GGPP and FPP^69,70^. Transcriptomic data showed that AZ-4 had four upregulated and 20 downregulated WRKY TFs, while CAL-2 had two upregulated and five downregulated WRKY TFs. Only one downregulated WRKY TF was expressed the two cultivars (Fig. 8C).

### Aquaporins expression under drought response analysis

Aquaporins are integral membrane proteins that facilitate water transport across cell membranes, playing a critical role in plant water balance, development, and drought adaptation. Since the majority of these proteins tend to be downregulated under drought conditions, reducing water permeability to conserve water, we investigated transcripts that were annotated as aquaporins in our data sets for AZ-4 and CAL-2 between drought and irrigated conditions. We found a total of 23 transcripts in AZ-4 and 26 transcripts in CAL-2 that were annotated as aquaporins via at least one of the annotation databases such as NR, NT, PFAM, KO, Swiss-Prot, GO, and KOG. There are 15 transcripts that overlapped between the two cultivars’ comparisons (highlighted in Table 1). For AZ-4, there were 2 transcripts that were significantly upregulated, with log2 fold change of + 7.39 (homolog of PIP1-2) and + 2.48 (homolog of PIP1-4), while the rest were all significantly downregulated, with the most downregulated one is a TIP-type protein with − 6.55 for log2 fold change. For CAL-2, there were seven transcripts that were significantly upregulated, and the highest two transcripts, coded for TIP-type proteins, whose log2 fold change reached + 8.17, and a homolog of NIP5-1 whose log2 fold change was + 2.19. Meanwhile, the other transcripts were significantly downregulated, with the most downregulated is homolog of TIP1-2 with log2 fold change to be − 4.24.

## Discussion

### Transcriptome assembly and differential analysis for guayule’s drought stress tolerance

Differential expression analysis revealed distinct transcriptomic responses to drought and irrigation in both AZ-4 and CAL-2. A total of 6,167 transcripts were differentially expressed in AZ-4 and 5,396 in CAL-2. The contrasting proportions of up- and downregulated genes suggest differing stress adaptation strategies. Notably, comparisons between AZ-4 and CAL-2 under stress conditions revealed significant transcriptomic divergence with 15,275 transcripts. Functional annotation across seven databases highlighted the breadth of transcriptional activity. Over 50% and 45% of transcripts aligned with the NR and NT databases, respectively, offer robust protein and nucleotide mappings. PFAM and KEGG annotations reflected key protein families and metabolic pathways, while high representation in Swiss-Prot and GO highlighted functional diversity. The lower KOG coverage pointed to specific eukaryotic orthologous groups relevant to downstream analysis. Together, these results confirm the dataset’s utility in exploring functional responses to environmental stress.

Transcription factor analysis identified 2,929 transcripts across 69 TF families, emphasizing their regulatory importance in guayule’s drought adaptation. MYB and WRKY TFs were notable for roles in osmotic adjustment and stomatal regulation. In oilseed crops, these TFs enhance antioxidant activity and osmoprotectant synthesis, indicating conserved stress-response functions^46,74,75^. C2H2 and AP2/ERF TFs, including DREBs, activated stress-responsive genes via hormone signaling and water conservation^24,76,77^. bHLH TFs regulated genes involved in drought tolerance and secondary metabolite biosynthesis, crucial for protective compound and rubber production^78,79^. These findings highlight the diversity and critical roles of TFs in guayule’s stress tolerance. Current GO enrichment analysis revealed both shared and unique transcript functions in response to drought stress in guayule. In AZ-4, expressing genes controlling ‘carbohydrate metabolic process’ points to energy storage and biomass maintenance during drought stress, supporting rubber biosynthesis. In CAL-2, expressing genes controlling ‘transporter activity’ suggests efficient resource allocation under stress. Among three overlapping sub-categories for gene, gene families, and functions, ‘catalytic activity’ and ‘oxidoreductase activity’ were crucial for biochemical reactions and ROS mitigation. Exclusive sub-categories like ‘cyclase activity’ and ‘histone binding’ in AZ-4 indicate broader regulatory adjustments, while CAL-2’s ‘nitrogen cycle metabolic process’ suggests targeted metabolic adaptations. This reflects AZ-4’s emphasis on energy and growth, and CAL-2’s focus on resource management. In current study, drought stress significantly impacts key cellular processes in guayule, regardless of the genotypes. Repression of ‘carbohydrate metabolism’ and enzymatic activities like ‘catalytic’, ‘hydrolase’, ‘oxidoreductase’, and ‘transferase’ hinders energy production and metabolic balance. Downregulated processes such as ‘cell wall organization’, the ‘extracellular matrix’, and ‘cytoskeletal dynamics’, like ‘microtubule movement’, ‘cytoskeletal protein binding’, ‘motor activity’ reduce structural integrity, intracellular transport, and flexibility. Additionally, suppressed ‘antioxidant activity’ and certain aspects of ‘oxidoreductase activity’ reflect the plant’s effort to conserve energy, limiting its oxidative stress response. Together, these constraints affect plant growth, structure, and stress responses^80–82^. Conversely, drought stress also triggers the upregulation of critical adaptive processes. Also, it was found that ‘oxidoreductase activity’ in multiple comparisons, indicating that oxidative stress may need to be finely regulated rather than following a simple on-or-off response, as enhanced ‘oxidoreductase activity’ supports the management and mitigation of oxidative stress. Increased ‘lyase activity’ promotes metabolic flexibility, while heightened ‘transporter activity’ and ‘transmembrane transport’ ensure efficient resource allocation. The upregulation of ‘lipid metabolism’ strengthens membranes and signaling, and processes like ‘sulfur compound’ and ‘amino acid metabolism’ boost detoxification and stress response^83^. These adaptations emphasize guayule plant’s strategic focus on tolerance and survival under drought stress.

KEGG pathway analysis reveals distinct drought-response strategies among guayule cultivars (detailed and mentioned in Sect.  2.4). Shared pathways like ‘plant hormone signal transduction’, ‘circadian rhythm – plant’, and ‘starch and sucrose metabolism’ highlight conserved mechanisms for water conservation, stress timing, and energy mobilization. AZ-4 uniquely enriches pathways such as ‘glycosphingolipid biosynthesis,’ ‘glucosinolate biosynthesis,’ and ‘ribosome biogenesis,’ emphasizing its focus on cellular growth and maintenance. Interestingly, these same pathways were previously identified in tea plants Chen, et al.^69^, suggesting potential functional similarities between guayule and tea plants. Meanwhile, CAL-2 focuses on external defense through ‘anthocyanin biosynthesis’ and antibiotic-related pathways. These pathways closely align with findings compiled by Dabravolski and Isayenkov^84^ in their review of anthocyanin functions across multiple plant species, suggesting that CAL-2 employs similar strategies for stress resistance and environmental adaptation. In pooled samples, downregulated pathways, such as ‘plant hormone signal transduction’, ‘phenylpropanoid biosynthesis’, ‘pentose and glucuronate interconversions’, ‘starch and sucrose metabolism’, and ‘phagosome’ activity, all reflect conserved strategies of reduced signaling, metabolic activity, and structural investment to conserve energy. Suppressed processes like ‘phenylalanine metabolism’ in AZ-4 and ‘cyanoamino acid metabolism’ in CAL-2 show genotype-specific adjustments aimed at minimizing energy use. Upregulated pathways emphasize enhanced drought tolerance. ‘Circadian rhythm – plant’ optimizes biological timing, while ‘galactose metabolism’ and ‘glycerolipid metabolism’ support energy and membrane stability, similar to what was found in *Dendrobium nobile* Lv, et al.^85^. AZ-4 shows increased ‘glyoxylate and dicarboxylate metabolism’ and ‘glutathione metabolism’ for detoxification and stress mitigation. CAL-2 enriches ‘alpha-linolenic acid metabolism’, ‘linoleic acid metabolism’, and ‘cysteine and methionine metabolism’, enhancing signaling and sulfur-based stress responses, with ‘valine’, ‘leucine’, and ‘isoleucine degradation’ boosting metabolic flexibility, similar to *Dendrobium nobile* Lv, et al.^85^, and *Stephania kwangsiensis* Huang, et al.^86^. Together, these pathways reflect guayule’s proactive, genotype-specific strategies to endure drought conditions.

Overall, RNA-seq analysis reveals that AZ-4 and CAL-2 employ distinct yet complementary strategies to cope with drought stress. While AZ-4 prioritizes internal resource management, growth, and structural maintenance, CAL-2 emphasizes external defense and efficient resource redistribution. Despite some shared core stress-response pathways, the accessions exhibit divergent transcriptomic and regulatory profiles that reflect their unique adaptive mechanisms.

### Shared and unique drought upstream responses between AZ-4 and CAL-2

The findings reveal notable differences in transcriptomic responses between AZ-4 and CAL-2 plants grown under drought stress. AZ-4 exhibited a higher proportion of significantly upregulated annotated TFs (96.4% vs. CAL-2’s 84.7%), along with a greater number of downregulated TF (255 transcripts vs. 139 transcripts). This suggests AZ-4 engages in broader transcriptional reprogramming to adapt to drought stress. In contrast, CAL-2 showed fewer transcriptomic changes, implying a strategy focused more on proteomic adjustments, favoring translation and deployment of specific proteins over widespread gene expression shifts. Despite these differences, both cultivars shared 30 upregulated and 97 downregulated TFs, pointing to some conserved regulatory responses. The three most abundant TF families identified were AP2/ERF, MYB, and NAC.

Several AP2/ERF (ERF) genes showed significant upregulated expression changes under drought stress, including homologs of DREB2A, DREB2B, DREB2C, ERF1B, ERF34, ERF40, and ERF105 (annotated via mentioned databases in 2.3). This aligns with published studies on members of the DREB family (DREB2A, DREB2B, DREB2C) activate downstream stress-responsive genes^87–90^. Other ERFs, such as ERF34 and ERF40, are known to promote lignin biosynthesis and cell wall modification, enhancing structural resilience under stress in rice and *Arabidopsis*, respectively^91,92^. ERF1B and ERF105, typically associated with cold in *Plumbago indica*^93^ and fungal responses in *Arabidopsis*^94–96^, were upregulated and may participate in drought-related crosstalk. Conversely, several ERFs exhibited significantly downregulated expression under drought stress, including ERF5, ERF6, ERF14, ERF22, ERF36, ERF59, ERF71, and ERF72. Reduced expression of these genes likely contributes to energy conservation, as they are known to modulate root architecture, inhibit excessive elongation, and prioritize survival over growth during drought^76,97–99^.

Expression changes in MYB genes reflect distinct drought tolerance strategies. Upregulated MYBs, including homologs of MYB48, MYB59, MYBS2, and MYBS5, play critical roles in drought stress tolerance. These transcription factors may be particularly beneficial, as MYB48 and MYB59 are involved in delaying senescence and reinforcing structural integrity^100,101^, while MYBS2 and MYBS5 contribute to sugar metabolism and osmotic regulation^102^. Additionally, clock-related genes such as LHY1 and RVEs (RVE1, RVE8) were also upregulated, potentially aiding in the coordination of stress timing through circadian rhythm regulation^46,103^. In contrast, downregulated MYBs, including MYB24, MYB44, MYB46, MYB91, and KUA1, may facilitate drought tolerance via distinct mechanisms. For example, reduced expression of MYB91, MYB44, and MYB24 has been linked to balanced antioxidant responses and improved osmotic balance^46,104^, while lower levels of MYB46 and KUA1 may help with tissue flexibility, water-use efficiency, and ROS accumulation and signaling in *Betula platyphylla*^105^ and tomato^106^.

Expression changes in NAC transcription factors underscore key drought adaptation strategies. Upregulated NACs, including NAC019, NAC072, and NAC102, are critical regulators of ABA and JA signaling pathways that enhance stress responses and drought tolerance^107–109^. In contrast, downregulated NAC genes: NAC01, NAC03, NAC043, NAC086, NAC083, and NAC090, may suggest a shift away from growth toward stress mitigation. These genes are typically associated with growth, cell wall biosynthesis, and oxidative stress protection, and may have reduced activity to prioritize survival mechanisms^110–114^. Downregulating NAC01 and NAC03, previously found to be drought positive regulators in other plant species^115,116^, may appear counterintuitive, this adjustment likely represents a trade-off favoring more efficient or species-specific drought tolerance strategies in guayule.

Altogether, these patterns suggest that AZ-4 favors extensive transcriptional regulation, whereas CAL-2 may rely on more limited transcriptional regulation or possibly post-transcriptional mechanisms. The prominence of AP2/ERF, MYB, and NAC transcription factor families in the RNA-Seq analysis is a rational finding, as these families are well-established regulators of drought stress responses in plants^117,118^. Similar patterns have been reported in other studies, further supporting their roles in orchestrating key mechanisms for drought tolerance using guayule cultivar AZ2^119^. Furthermore, this study represents the first instance of two distinct guayule cultivars being sampled and analyzed side by side. Therefore, the shared and distinct TF responses observed between the two highlights both convergence and divergence in their evolutionary adaptations to drought.

### Insights from the prominent six TFs for MVA and MEP pathways under drought

**Fig. 9:**
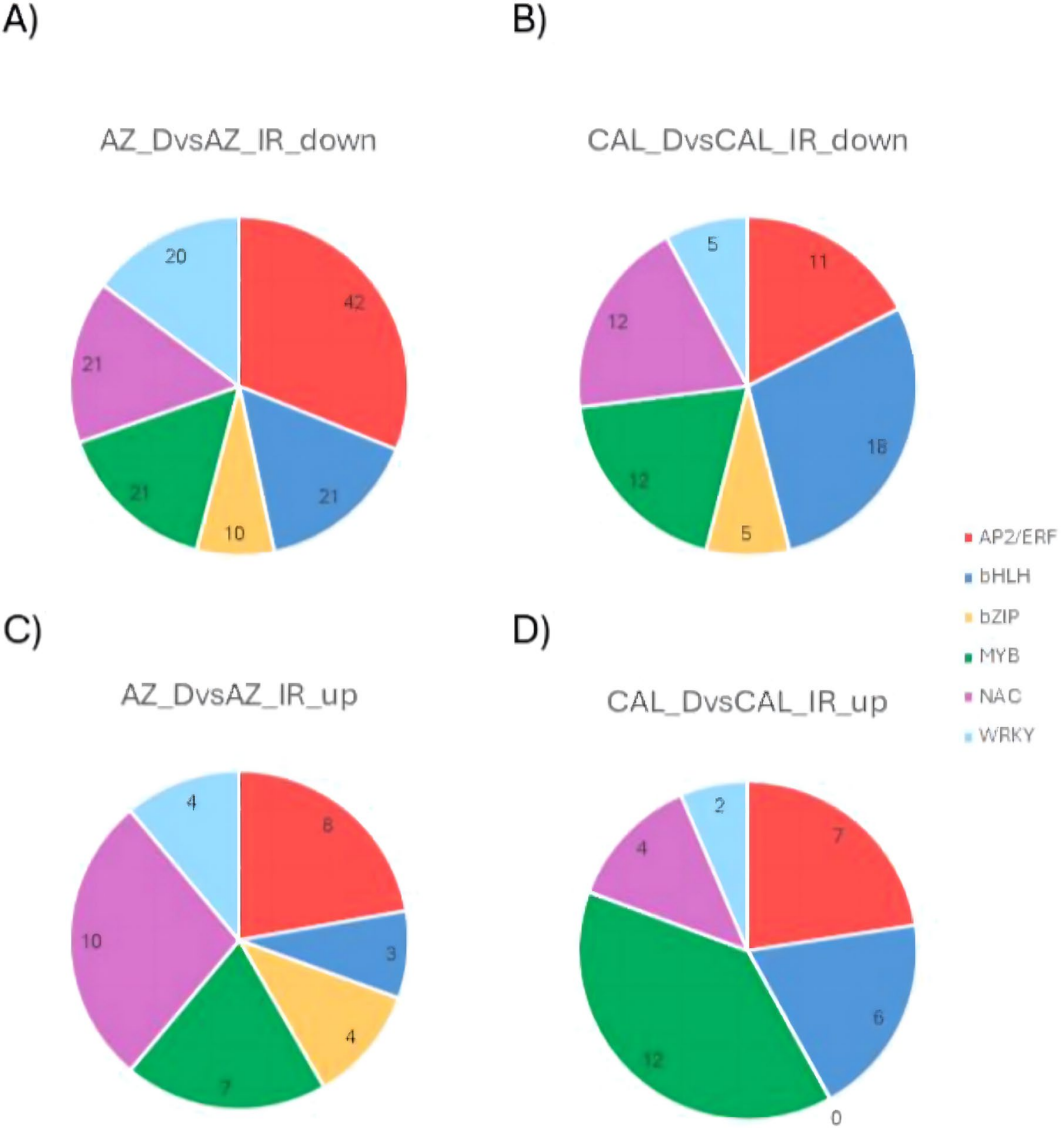
Distribution profiles of the six regulatory TF families for enzymes in the MVA and MEP pathways under drought. (**A**) Profile for downregulated comparison between AZ-4 drought and irrigated samples. (**B**) Profile for downregulated comparison between CAL-2 drought and irrigated samples. (**C**) Profile for upregulated comparison between AZ-4 drought and irrigated samples. (**D**) Profile for upregulated comparison between CAL-2 drought and irrigated samples. Red: AP2/ERF; Blue: bHLH; Yellow: bZIP; Green: MYB; Purple: NAC; Teal: WRKY.

The overlap in transcription factors (TFs) suggests potentially distinct regulatory strategies, as AZ-4 exhibited five times more AP2/ERF TFs being downregulated (Fig. 9A, B). This indicates deeper repression at the early steps of the MVA and MEP pathways, fixing acetyl-CoA and synthesizing mevalonate, and synthesizing HMBPP from G3P and pyruvate, respectively. Further downstream, the AP2/ERF family also regulates IDI, GGPPS, FPPS, and TPS, which are essential for IPP production and subsequent mono-, di-, and triterpene biosynthesis. The higher number of downregulated AP2/ERF TFs in AZ-4 (42 down vs. eight up) may contribute to reduced natural rubber biosynthesis, similar to other studies using AZ-3 cultivar^5^. In contrast, CAL-2’s more limited response (11 down vs. seven up; Fig. 9C, D) reflects differences in drought stress adaptation between the genotypes. This divergence of approximately four times fewer TFs in CAL-2, highlights the distinct regulatory mechanisms for rubber biosynthesis in both cultivars under drought.

The bHLH transcription factor family, well-known for its roles in regulating plant growth, development, and stress responses, showed a higher number of downregulated than upregulated TF genes in both guayule cultivars, AZ-4 (21 downregulated vs. three upregulated) and CAL-2 (18 downregulated vs. six upregulated). Given that bHLH transcription factors serve as key regulators at both the early and late steps of the MVA and MEP pathways in rubber tree, examining how these TFs are regulated in the two cultivars is crucial to understanding their influence on terpenoid biosynthesis under drought stress^44^. Interestingly, both cultivars appear to adopt a convergent strategy for the bHLH family, as the number of downregulated genes in AZ-4 (21) and CAL-2 (18) are relatively similar (Fig. 9A, B). However, some bHLH genes were upregulated. These upregulated genes may help with triggering drought responses and regulating stress adaptation mechanisms. Overall, this suggests that both cultivars exhibit convergent utilization of the bHLH TF family for reducing rubber biosynthesis and stress responses during drought conditions.

AZ-4’s drought response includes both up- and downregulation of bZIP TFs (10 down vs. 4 up), activating genes in the MVA and MEP pathways while also conserving resources or redirecting metabolism. CAL-2, however, displayed uniform suppression of bZIP TFs (five downregulated vs. none upregulated; Fig. 9B, D), indicating a stronger emphasis on limiting natural rubber biosynthesis). These patterns suggest AZ-4 employs a more dynamic regulatory approach, while CAL-2 focuses on metabolic conservation with no bZIP TFs getting upregulated. The shared downregulation of bZIP TFs suggests conserved core drought-response mechanisms. However, the overall regulatory strategies differ, as no bZIP genes are upregulated in CAL-2. This difference highlights unique transcriptomic adjustments for rubber biosynthesis under drought in each cultivar.

The overlap in downregulated NAC TFs between AZ-4 and CAL-2 suggests shared stress responses, yet AZ-4 demonstrated broader transcriptional shifts (21 down vs. 10 up; Fig. 9A, C) with more about double the numbers of TFs being both up- and downregulated than CAL-2 (12 down vs. four up; Fig. 9B, D). This indicates a more flexible adaptation strategy in AZ-4, potentially enabling broader metabolomic adjustments under drought conditions to lower rubber biosynthesis^5^. In contrast, CAL-2 showed stricter and fewer changes in NAC TFs, pointing to a more stable regulatory framework focused on resource preservation, with minimal alterations to this regulatory family involved in rubber biosynthesis pathways. These differences highlight different strategies of rubber biosynthesis and drought tolerance in the two guayule cultivars.

AZ-4 downregulated more MYB TFs (21 down vs. 7 up; Fig. 9A, C), potentially prioritizing resource conservation by lowering MVA and MEP pathway fluxes and reducing precursor synthesis. Meanwhile, CAL-2 maintained a more balanced MYB profile (12 down vs. 12 up; Fig. 9B, D), supporting a stable response under stress. The shared upregulated MYB TFs in both cultivars may play conserved roles in sustaining the enzymatic activity of upstream elements of terpenoid biosynthesis during drought, highlighting overlapping mechanisms amid broader regulatory differences of the two guayule cultivars under drought.

WRKY transcription factors play a crucial role in the MVA and MEP pathways^42,120^. AZ-4 exhibited significant downregulation of WRKY genes (20 down vs. 4 up), a reduction four times greater than CAL-2’s impact (5 down vs. 2 up). This suggests that AZ-4 suppressed the synthesis of stress-related secondary metabolites, as WRKY TFs typically regulate these compounds in response to stress^120^. In contrast, CAL-2 showed minimal WRKY changes, indicating a more restrained, finely tuned, and stable response. The limited overlap between the two cultivars, sharing only a single downregulated WRKY transcript – WRKY70, highlights their divergence in WRKY-mediated rubber biosynthesis, likely influenced by genetic factors or differing environmental conditions. These distinct regulatory patterns may reflect differences in how AZ-4 and CAL-2 manage cellular metabolism and rubber production under drought stress.

**Fig. 10:**
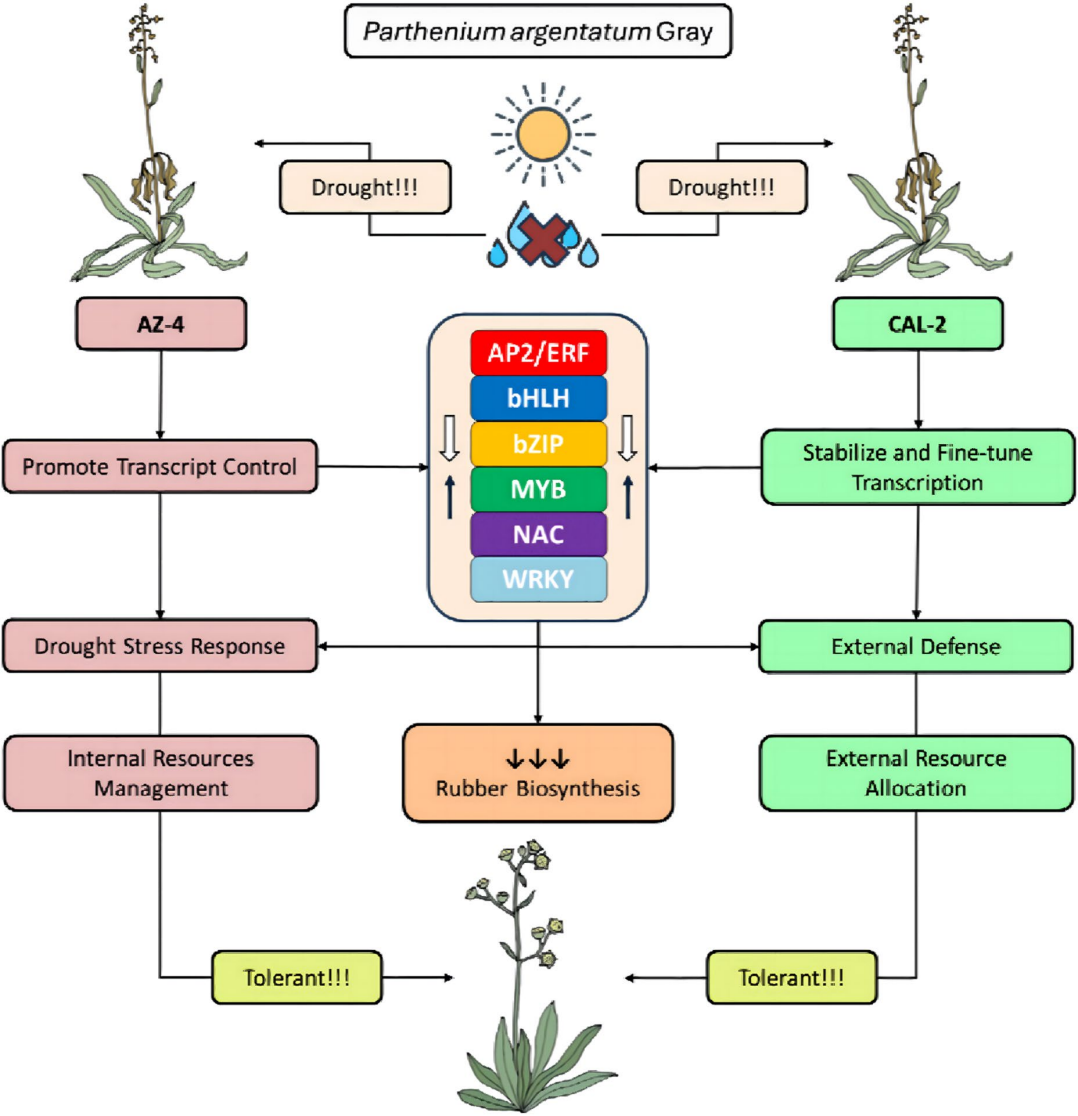
Proposed model for the molecular mechanisms of guayule (*P. argentatum* Gray) cultivars AZ-4 and CAL-2 for rubber biosynthesis and drought tolerance. AZ-4 emphasizes flexibility y, broad metabolic engagement, and internal resource management, while CAL-2 focuses on stability, precise regulatory control, and external defense. AZ-4’s adaptive approach enhances stress tolerance through amplified transcriptional activity, whereas CAL-2 relies on fine-tuned adjustments to maintain resilience.

This study provides an initial examination of how two distinct guayule cultivars respond to drought conditions. Furthermore, it is the first to focus on the regulation of the six major TF families: AP2/ERF, bHLH, bZIP, MYB, NAC, and WRKY, with reduction of transcripts from all six families could potentially help explain the lowering of rubber biosynthesis in AZ-4, and all except WRKY helped explain the lowering in CAL-2. Interestingly, both cultivars share core drought responses, such as limited overlap in upregulated TFs (Fig. 9C, D) and numerous downregulated ones (Fig. 9A, B), suggesting conserved strategies to suppress terpenoid pathways and slow natural rubber biosynthesis. However, AZ-4 exhibits a more dynamic response, with broader TF regulation compared to CAL-2’s predominantly suppressive approach (Fig. 10). By uncovering the unique regulatory networks and response strategies employed by AZ-4 and CAL-2, this paper provides valuable insights into how guayule adapts its cellular metabolism and secondary metabolite synthesis to drought.

### Aquaporin transcriptomic profiles showed potential reduction in water uptake in guayule

Aquaporins are important for regulating cellular water transport, particularly in response to environmental stressors such as drought. They play a critical role in maintaining cellular water balance, which is essential for sustaining metabolic processes during drought stress. This study found that the majority of guayule aquaporin transcripts were significantly downregulated in response to drought stress (21/23 aquaporins were downregulated in AZ-4, 19/26 for CAL-2), consistent with findings documented in other reviews^121,122^. A subset of aquaporin transcripts showed significant upregulation, such as PIP1-2 and PIP1-4 in AZ-4, and NIP5-1 in CAL-2. Their expression is highly sensitive to water availability, making aquaporin regulation a reliable molecular proxy for confirming drought conditions in transcriptomic analyses. The selective upregulation of these aquaporins suggests potential compensatory mechanisms, where certain isoforms may be activated to maintain minimal water and nutrient transport despite overall aquaporin downregulation. Interestingly, PIP1-4 has been found to transport hydrogen peroxide, which acts as a signaling molecule in stress responses^123^. Hence, it is possible that PIP1-4 got upregulated to help guayule build up stress molecules for initiating and maintaining drought response signaling. Moreover, NIP5-1 is primarily known for its role in boron transport, which is important for cell wall integrity and membrane stability^124^. Thus, upregulation of NIP5-1 may help ensure minimal boron uptake to support cell structure and function, as under drought conditions, boron availability fluctuates^125^. Transcription factors control aquaporin gene expression, thereby modulating water transport efficiency and helping plants adapt to water limitation. Importantly, the aquaporin expression patterns in this study for PIP1-2 and PIP1-4 that is in agreement with previous studies^5,56^ whom demonstrated upregulation of PIP1-3 and PIP2s in guayule. This regulatory relationship indirectly affects natural rubber biosynthesis, as terpenoid production via the MVA and MEP pathways depends on stable cellular hydration and energy balance. Overall, this study provides insight into aquaporin regulation during drought in different guayule cultivars. Through coordinated control of aquaporins, transcription factors help preserve the physiological environment necessary for rubber biosynthesis under drought stress.

### Future directions

Future research should expand beyond transcriptomics to directly measure how regulatory differences influence natural rubber production. Extracting and quantifying rubber yield from AZ-4 and CAL-2 under both drought and irrigated conditions would connect molecular findings to practical outcomes.

Our data aligned with Luo and Abdel-Haleem^10^ who reported that guayule accessions collected from diverse geographic regions, such as Arizona, California, Texas and Mexico showed a wide array for responses to drought stress conditions. This could be an indicator of evolving different drought stress tolerance mechanisms in USDA guayule collection. The scope of future studies could be broadened to include guayule genotypes from diverse geographic regions. This approach would help identify wider drought stress tolerance mechanisms, as well conserved and region-specific adaptive genes. Understanding the effect of drought, at the molecular level, would also allow comparative studies among natural rubber harboring species. Proteomic and metabolomic analyses would complement these efforts, capturing changes in protein abundance and metabolite profiles that are crucial for drought adaptation and terpenoid biosynthesis. Such studies could provide deeper insight into the biochemical mechanisms driving resource allocation and stress resilience. By combining transcriptomics, proteomics, metabolomics, and environmental stress studies, researchers can unravel the complex regulatory networks governing guayule’s stress responses. Functional validation of transcription factors, through gene-editing or overexpression experiments, could pinpoint the most effective regulators of the MVA and MEP pathways. These efforts will guide breeding strategies aimed at maximizing natural rubber yield while ensuring resilience in the face of dry weather, and accelerate the development of drought-tolerant guayule cultivars optimized for different growing zones.

## Conclusion

This study provides a focused analysis of transcriptional regulation in guayule cultivars AZ-4 and CAL-2 under drought stress, revealing distinct adaptive strategies of both cultivars, for the first time. AZ-4 exhibits a flexible, growth-oriented response characterized by broad transcriptional shifts and amplified stress-regulation, while CAL-2 maintains a more stable and targeted defense strategy, emphasizing resource management and external protection. Despite these differences, both accessions share core stress-response mechanisms, particularly in terpenoid biosynthesis via the MVA and MEP pathways. Differential expressions of key transcription factor families, including AP2/ERF, MYB, NAC, WRKY, bHLH, and bZIP, highlights accession-specific regulatory patterns, with drought-specific modulation observed in bHLH and bZIP. Aquaporin expression further underscores the role of water balance regulation, suggesting selective upregulation as a potential mechanism for maintaining physiological function under stress. Together, these findings enhance our understanding of guayule’s drought tolerance and offer a foundation for future breeding and genetic engineering strategies aimed at improving natural rubber production in arid regions of the southwestern United States.

## Supplementary Information

Below is the link to the electronic supplementary material.

Supplementary Material 1

Supplementary Material 2

Supplementary Material 3

## Data Availability

The datasets used and/or analyzed during the current study are included in the manuscript and raw RNA-Seq data has been submitted to NCBI Sequence Read Archive database and publicly available. The Submission ID is SUB15929463; the Bio Project ID is PRJNA1415201; and the SRA accessions are SRR37002812, SRR37002809, SRR37002806, SRR37002803, SRR37002811, SRR37002808, SRR37002805, SRR37002802, SRR37002810, SRR37002807, SRR37002804 and SRR37002801; and the access link: https://www.ncbi.nlm.nih.gov/bioproject/1415201.
