# Supplementary material for: Expression profiles of transcription factors and aquaporins suggest putative roles in rubber biosynthesis regulation and drought stress adaptation in guayule
Source: Sci Rep. 2026 Apr 7;16:11718. doi: 10.1038/s41598-026-44868-9 (PMC13061947; doi:10.1038/s41598-026-44868-9)
Supplement: Supplementary file 1 — Supplementary Material 1 [file 41598_2026_44868_MOESM1_ESM.docx]

**Supplementary Table 1. Detailed mapping statistics per sample.**

| sample | library | raw_reads | raw_bases | clean_reads | clean_bases | error_rate | Q20 | Q30 | GC_pct |
| --- | --- | --- | --- | --- | --- | --- | --- | --- | --- |
| AZ_IR_r1_1 | CRWS230006108-1a | 30193585 | 9.1 | 29888238 | 9 | 0.03 | 97.83 | 93.58 | 43.04 |
| AZ_IR_r2_2 | CRWS230006109-1a | 36096841 | 10.8 | 35721493 | 10.7 | 0.03 | 97.79 | 93.51 | 43.28 |
| AZ_IR_r3_3 | CRWS230006110-1a | 39424058 | 11.8 | 38969501 | 11.7 | 0.03 | 97.79 | 93.52 | 43.15 |
| CAL_IR_r1_4 | CRWS230006111-1a | 38594027 | 11.6 | 38165458 | 11.4 | 0.03 | 97.66 | 93.14 | 43.25 |
| CAL_IR_r2_5 | CRWS230006112-1a | 47219768 | 14.2 | 46758983 | 14 | 0.03 | 97.45 | 92.67 | 43.12 |
| CAL_IR_r3_6 | CRWS230006113-1a | 36269694 | 10.9 | 35738332 | 10.7 | 0.03 | 97.53 | 92.86 | 43.54 |
| AZ_D_r1_7 | CRWS230006114-1a | 51758262 | 15.5 | 50856868 | 15.3 | 0.03 | 97.48 | 92.75 | 43.13 |
| AZ_D_r2_8 | CRWS230006115-1a | 32878204 | 9.9 | 32353320 | 9.7 | 0.03 | 97.54 | 92.89 | 43.56 |
| AZ_D_r3_9 | CRWS230006116-1a | 34397440 | 10.3 | 33923577 | 10.2 | 0.03 | 97.53 | 92.88 | 43.29 |
| CAL_D_r1_10 | CRWS230006117-1a | 36902033 | 11.1 | 36489655 | 10.9 | 0.03 | 97.53 | 92.84 | 43.22 |
| CAL_D_r2_11 | CRWS230006118-1a | 33145932 | 9.9 | 32774839 | 9.8 | 0.03 | 97.76 | 93.42 | 43.68 |
| CAL_D_r3_12 | CRWS230006119-1a | 40197850 | 12.1 | 39704152 | 11.9 | 0.03 | 97.4 | 92.57 | 43.67 |

**Supplementary Table 2. Significant GO terms found in five different comparisons between guayule cultivars AZ-4 and CAL-2.**

| **Data set** | **Description** | **Adjusted p-value** | **Gene count** |
| --- | --- | --- | --- |
| AZ_DvsAZ_IR_DOWN | carbohydrate metabolic process | 5.50E-16 | 206 |
|  | catalytic activity | 4.72E-15 | 1356 |
|  | cell wall | 9.63E-08 | 26 |
|  | transferase activity | 1.82E-07 | 608 |
|  | external encapsulating structure | 6.67E-07 | 34 |
|  | cell wall organization or biogenesis | 8.02E-07 | 39 |
|  | hydrolase activity | 2.28E-05 | 525 |
|  | microtubule-based movement | 7.94E-05 | 27 |
|  | catalytic activity, acting on a protein | 0.000124 | 440 |
|  | motor activity | 0.001314 | 29 |
|  | cytoskeletal protein binding | 0.001314 | 60 |
|  | oxidoreductase activity | 0.001522 | 262 |
|  | antioxidant activity | 0.002042 | 30 |
|  | protein modification process | 0.004423 | 318 |
| CAL_DvsCAL_IR_DOWN | carbohydrate metabolic process | 8.67E-12 | 144 |
|  | catalytic activity | 6.14E-07 | 901 |
|  | cell wall organization or biogenesis | 1.91E-06 | 31 |
|  | hydrolase activity | 2.25E-06 | 377 |
|  | microtubule-based movement | 1.25E-05 | 23 |
|  | motor activity | 1.25E-05 | 27 |
|  | cell wall | 1.45E-05 | 18 |
|  | antioxidant activity | 7.16E-05 | 27 |
|  | external encapsulating structure | 0.000329 | 22 |
|  | cytoskeletal protein binding | 0.001161 | 45 |
|  | oxidoreductase activity | 0.001161 | 188 |
|  | transmembrane transport | 0.008108 | 161 |
|  | cytoskeleton | 0.021993 | 55 |
|  | transporter activity | 0.04421 | 163 |
| DvsIR_DOWN | carbohydrate metabolic process | 2.18E-19 | 183 |
|  | catalytic activity | 4.19E-11 | 1066 |
|  | hydrolase activity | 5.60E-10 | 455 |
|  | cell wall | 1.98E-08 | 24 |
|  | microtubule-based movement | 2.47E-07 | 28 |
|  | external encapsulating structure | 4.78E-07 | 30 |
|  | motor activity | 4.78E-07 | 32 |
|  | cell wall organization or biogenesis | 1.95E-06 | 33 |
|  | cytoskeletal protein binding | 2.07E-05 | 56 |
|  | antioxidant activity | 0.000302 | 28 |
|  | cytoskeleton | 0.000469 | 70 |
|  | extracellular matrix organization | 0.009449 | 4 |
|  | catalytic activity, acting on a protein | 0.010925 | 335 |
|  | transferase activity | 0.011086 | 447 |
|  | oxidoreductase activity | 0.014409 | 204 |
|  | molecular function regulator | 0.018685 | 94 |
| AZ_DvsAZ_IR_UP | oxidoreductase activity | 1.15E-06 | 126 |
|  | lyase activity | 0.010645 | 32 |
| CAL_DvsCAL_IR_UP | oxidoreductase activity | 6.38E-14 | 197 |
|  | transporter activity | 8.29E-08 | 163 |
|  | transmembrane transport | 3.85E-06 | 147 |
|  | lipid metabolic process | 0.000607 | 84 |
|  | sulfur compound metabolic process | 0.005554 | 26 |
|  | cellular modified amino acid metabolic process | 0.007385 | 24 |
|  | catalytic activity | 0.01547 | 660 |
|  | lyase activity | 0.044203 | 37 |
| DvsIR_UP | oxidoreductase activity | 2.02E-06 | 127 |
|  | lipid metabolic process | 0.000548 | 66 |

**Supplementary Table 3. Significant KEGG pathways found in five different comparisons between guayule cultivars AZ-4 and CAL-2.**

| **Data set** | **Description** | **Adjusted p-value** | **Gene count** |
| --- | --- | --- | --- |
| AZ_DvsAZ_IR_DOWN | Plant hormone signal transduction | 1.59E-11 | 79 |
|  | Phenylpropanoid biosynthesis | 5.63E-07 | 46 |
|  | Phagosome | 1.73E-05 | 32 |
|  | Pentose and glucuronate interconversions | 4.92E-05 | 25 |
|  | Starch and sucrose metabolism | 0.000851 | 42 |
|  | Phenylalanine metabolism | 0.035411 | 15 |
| CAL_DvsCAL_IR_DOWN | Pentose and glucuronate interconversions | 1.32E-05 | 22 |
|  | Plant hormone signal transduction | 4.93E-05 | 46 |
|  | Phagosome | 6.65E-05 | 24 |
|  | Phenylpropanoid biosynthesis | 6.65E-05 | 31 |
|  | Starch and sucrose metabolism | 0.002007 | 31 |
|  | Cyanoamino acid metabolism | 0.018111 | 11 |
| DvsIR_DOWN | Plant hormone signal transduction | 3.96E-10 | 64 |
|  | Pentose and glucuronate interconversions | 3.98E-06 | 24 |
|  | Phagosome | 2.51E-05 | 27 |
|  | Starch and sucrose metabolism | 9.11E-05 | 38 |
|  | Phenylpropanoid biosynthesis | 0.000244 | 32 |
|  | Cyanoamino acid metabolism | 0.046704 | 11 |
| AZ_DvsAZ_IR_UP | Circadian rhythm - plant | 2.60E-05 | 17 |
|  | Glyoxylate and dicarboxylate metabolism | 7.83E-05 | 20 |
|  | Glycerolipid metabolism | 7.97E-05 | 19 |
|  | Galactose metabolism | 0.000207 | 14 |
|  | Glutathione metabolism | 0.014168 | 14 |
| CAL_DvsCAL_IR_UP | Galactose metabolism | 1.96E-10 | 25 |
|  | Circadian rhythm - plant | 0.00223 | 15 |
|  | alpha-Linolenic acid metabolism | 0.00509 | 15 |
|  | Valine, leucine and isoleucine degradation | 0.025331 | 14 |
|  | Linoleic acid metabolism | 0.031091 | 7 |
|  | Cysteine and methionine metabolism | 0.03182 | 18 |
| DvsIR_UP | Galactose metabolism | 1.75E-10 | 23 |
|  | Circadian rhythm - plant | 1.31E-05 | 17 |
|  | Glycerolipid metabolism | 0.001017 | 17 |
|  | Glutathione metabolism | 0.001904 | 16 |
|  | Glyoxylate and dicarboxylate metabolism | 0.011776 | 15 |
|  | alpha-Linolenic acid metabolism | 0.044006 | 11 |

**Supplementary Figure 1. Transcription factor families regulating methylerythritol phosphate and mevalonate pathways in plants.** Six key transcription factor (TF) families: AP2/ERF, bHLH, bZIP MYB, NAC, and WRKY, regulate downstream enzymes in the methylerythritol phosphate (MEP) and mevalonate (MVA) pathways. Highlighted boxes with associated coloring are said families’ contributions to enzyme regulations. Blank boxes mean said families have little to no effect on corresponding enzyme’s activity.

**Supplementary Figure 2. Detailed read lengths and layout of Transcriptome sequencing and read assembly.** (A) length distribution of transcripts and unigene. (B) transcript length distribution. (C) unigene length distribution.
